# Time‐series multispectral imaging in soybean for improving biomass and genomic prediction accuracy

**DOI:** 10.1002/tpg2.20244

**Published:** 2022-08-22

**Authors:** Kengo Sakurai, Yusuke Toda, Hiromi Kajiya‐Kanegae, Yoshihiro Ohmori, Yuji Yamasaki, Hirokazu Takahashi, Hideki Takanashi, Mai Tsuda, Hisashi Tsujimoto, Akito Kaga, Mikio Nakazono, Toru Fujiwara, Hiroyoshi Iwata

**Affiliations:** ^1^ Graduate School of Agricultural and Life Sciences Univ. of Tokyo Tokyo Japan; ^2^ Arid Land Research Center Tottori Univ. Tottori Japan; ^3^ Graduate School of Bioagricultural Sciences Nagoya Univ. Nagoya Japan; ^4^ Faculty of Life and Environmental Sciences, Tsukuba Plant Innovation Research Center Univ. of Tsukuba Tsukuba Japan; ^5^ Soybean and Field Crop Applied Genomics Research Unit, Institute of Crop Science National Agriculture and Food Research Organization Tsukuba Japan

## Abstract

Multispectral (MS) imaging enables the measurement of characteristics important for increasing the prediction accuracy of genotypic and phenotypic values for yield‐related traits. In this study, we evaluated the potential application of temporal MS imaging for the prediction of aboveground biomass (AGB) in soybean [*Glycine max* (L.) Merr.]. Field experiments with 198 accessions of soybean were conducted with four different irrigation levels. Five vegetation indices (VIs) were calculated using MS images from soybean canopies from early vegetative to early reproductive stage. To predict the genotypic values of AGB, VIs at the different growth stages were used as secondary traits in a multitrait genomic prediction. The prediction accuracy of the genotypic values of AGB from MS and genomic data largely outperformed that of the genomic data alone before the flowering stage (90% of accessions did not flower), suggesting that it would be possible to determine cross‐combinations based on the predicted genotypic values of AGB. We compared the prediction accuracy of a model using the five VIs and a model using only one VI to predict the phenotypic values of AGB and found that the difference in prediction accuracy decreased over time at all irrigation levels except for the most severe drought. The difference in the most severe drought was not as small as that in the other treatments. Only the prediction accuracy of a model using the five VIs in the most severe droughts gradually increased over time. Therefore, the optimal timing for MS imaging may depend on the irrigation levels.

## Core Ideas

- Multispectral data are useful for predicting genotypic and phenotypic characteristics.
- We determined the timing for performing MS imaging to predict aboveground biomass.
- MS data before the flowering stage were useful for predicting the genotypic values of AGB.
- The optimal timing for MS imaging to predict AGB phenotypes depends on irrigation levels.
- These results will help decision making to determine the optimal timing for MS imaging.

**Abbreviations**
AGB: aboveground biomass
DTF: days to flowering
GEI: genotype × environment interaction
GRVI: green–red vegetation index
MS: multispectral
MT: multitrait
NDI_red_
: normalized difference index using red‐edge and red spectral reflectance
NDRE: normalized difference red‐edge index
NDVI: normalized difference vegetation index
PCA: principal component analysis
RTVI: red‐edge triangular vegetation index
SNP: single‐nucleotide polymorphism
UAV: unmanned aerial vehicle
VI: vegetation index

## 1 INTRODUCTION

Multispectral (MS) imaging can capture differences in plant growth status, such as chlorophyll content, disease status, and photosynthetic capacity, through differences in spectral reflectance (Gallo et al., 1985; Gitelson et al., 2003; Mahlein et al., 2013). By collecting MS imaging data and capturing plant growth status, we can predict important features such as future yield, biomass, and protein content (Hansen et al., 2002; Moges et al., 2004; Raun et al., 2001). Researchers have investigated the relationships between certain vegetation indices (VIs)—calculated based on spectral reflectance—and plant conditions (Chen et al., 2010; Delegido et al., 2011, 2013; Jorge et al., 2019; Motohka et al., 2010; Price & Bausch, 1995; Zarate‐Valdez et al., 2015). Multispectral image data and VIs have long been used in satellite remote sensing. Recently, small unmanned aerial vehicles (UAVs) equipped with MS cameras have been employed to collect high‐resolution MS image data from plants. The UAVs allow for the segmentation of pixels belonging to the subject (the plant) from those belonging to other objects (e.g., the background soil surface) (Jay et al., 2019; Jin et al., 2017; López‐Granados et al., 2016); thus, this method can sense certain conditions more accurately than satellite remote sensing (Khaliq et al., 2019). With the development of these technologies, it has become possible to use MS image data and VIs to evaluate the state of a plant and predict future growth. In soybean [*Glycine max* (L.) Merr.], the normalized difference vegetation index (NDVI), which is one of the VIs, has been reported to be useful in predicting grain yield from the flowering to the grain filling stage. (Christenson et al., 2016; Ma et al., 2001).

Multispectral image data are also used with genome‐wide marker data to predict certain traits (genomic prediction), as first demonstrated by (Rutkoski et al., 2016). The genetic correlation between MS image data and the target traits can be used to improve the prediction accuracy of genotypic values for said target traits. This approach is expected to improve selection accuracy for key breeding traits, such as yield, thus facilitating genetic gain. The use of such models, incorporating VIs as secondary traits collected during the vegetative and grain filling stages, has increased prediction accuracy for yield in maize (*Zea mays* L.) and wheat (*Triticum aestivum* L.) compared with the standard genomic prediction model (Sun et al., 2019, 2017).

It is sensible to imagine that MS imaging will be used in combination with remote sensing, such as UAVs, and other ‐omics technologies to improve the efficiency and accuracy of genetic improvement in crops. In this case, the timing of measurements is critical, as the phenological growth stages for a given crop will affect image data (Anche et al., 2020; Gnyp et al., 2013; Labus et al., 2002) including for soybean (Parmley et al., 2019). A previous study collected MS images three times from the late vegetative to the late reproductive stages and predicted the future phenotypic values of yield in soybean (Zhou et al., 2021). This study showed that MS images collected at the early and late reproductive growth stages is promising for estimating soybean yield. However, there are few studies in soybean that have predicted future genotype values for yield‐related traits based on MS images from early vegetative to early reproductive stages and evaluated their accuracy at each growth stage. Drought stress has also been reported to affect image data in soybean (Bi et al., 2018; Cure et al., 1989), suggesting that the level of genetic correlation between MS image data and the target trait is expected to vary depending on irrigation conditions. Multispectral imaging during the growth process will enable for the evaluation of changes in heritability and genetic and phenotypic correlations with target traits and eventually to identify the appropriate measurement time for predicting a given target trait. If genotype values for yield‐related traits can be predicted using MS data collected prior to flowering, promising cross‐combinations can be inferred based on the predicted genotype values. Such applications will contribute to increased efficiency and flexibility in soybean breeding.

**Core Ideas**

- Multispectral data are useful for predicting genotypic and phenotypic characteristics.
- We determined the timing for performing MS imaging to predict aboveground biomass.
- MS data before the flowering stage were useful for predicting the genotypic values of AGB.
- The optimal timing for MS imaging to predict AGB phenotypes depends on irrigation levels.
- These results will help decision making to determine the optimal timing for MS imaging.

In predicting wheat yield, a model using 62 reflectance bands in the 392–850 nm range acquired with a hyperspectral camera was more accurate than a model using only NDVI (Aguate et al., 2017). Aguate et al. (2017) found that the difference in the prediction accuracy between the two models decreased over time. However, these relationships are expected to vary according to irrigation treatments and crop species. If the timing and irrigation treatments at which other VIs, in addition to NDVI, improve the accuracy of phenotypic value prediction could be clarified, then the application of MS cameras would be expanded. In addition, improved accuracy in predicting the phenotypic values of target traits based on MS imaging would facilitate cultivation management and greatly help farmers in their decision making. Based on the predicted values, farmers may be able to irrigate more efficiently and predict yield earlier.

A high correlation has been reported between aboveground biomass (AGB) and soybean yield (Board et al., 1996; Ramírez‐Oliveras et al., 1997; Rao et al., 2002). In Japan, Korea, and China, the bean bug (*Riptortus pedestris*) causes severe damage to soybean, resulting in reduced yield (Bae et al., 2014; Li et al., 2019; Rahman & Lim, 2017). In our experimental field, because we applied pesticides three times before the destructive measurement of AGB, there was little damage on AGB. After the measurement of AGB, soybean yield was measured for the other plots, but the damage caused by bean bug feeding made it difficult to accurately measure soybean yields. Therefore, in this study, AGB was used as a target trait to reduce the effects of bug feeding. We collected MS imaging data from the early vegetative to the early reproductive stages of 198 soybean accessions grown under different irrigation levels. Drought stress generally causes severe damage to crop growth and ultimately lowers yield. Soybean, which serves as a major source of plant‐derived protein and oil (Sharma et al., 2014), exhibits a 40% reduction in yield because of drought on the global scale (Specht et al., 1999). By clarifying the genotypic relationships between AGB and MS image data, we may be able to use MS imaging for the genetic improvement of drought tolerance in soybean. If the variation in AGB with irrigation volume can be captured by MS imaging, efficient water‐saving agricultural practices using MS imaging will be possible.

We aimed to estimate the genetic correlations between five VIs and AGB in different irrigation levels over plant growth. In addition, we tested the extent to which the use of MS data before the flowering period could improve the prediction accuracy of the genotypic values for AGB. We also compared the accuracy of predicting AGB phenotypic values in a model using five VIs, including NDVI and a model using only NDVI, to evaluate the usefulness of the four VIs other than NDVI. Lastly, we predicted the phenotypic values of AGB using the correlation between VIs and AGB across all treatment conditions, thus evaluating whether macro‐environmental variability (i.e., the differences in irrigation levels) is reflected in the VIs.

## 2 MATERIALS AND METHODS

### 2.1 Experimental setup and data collection

**FIGURE 1:**
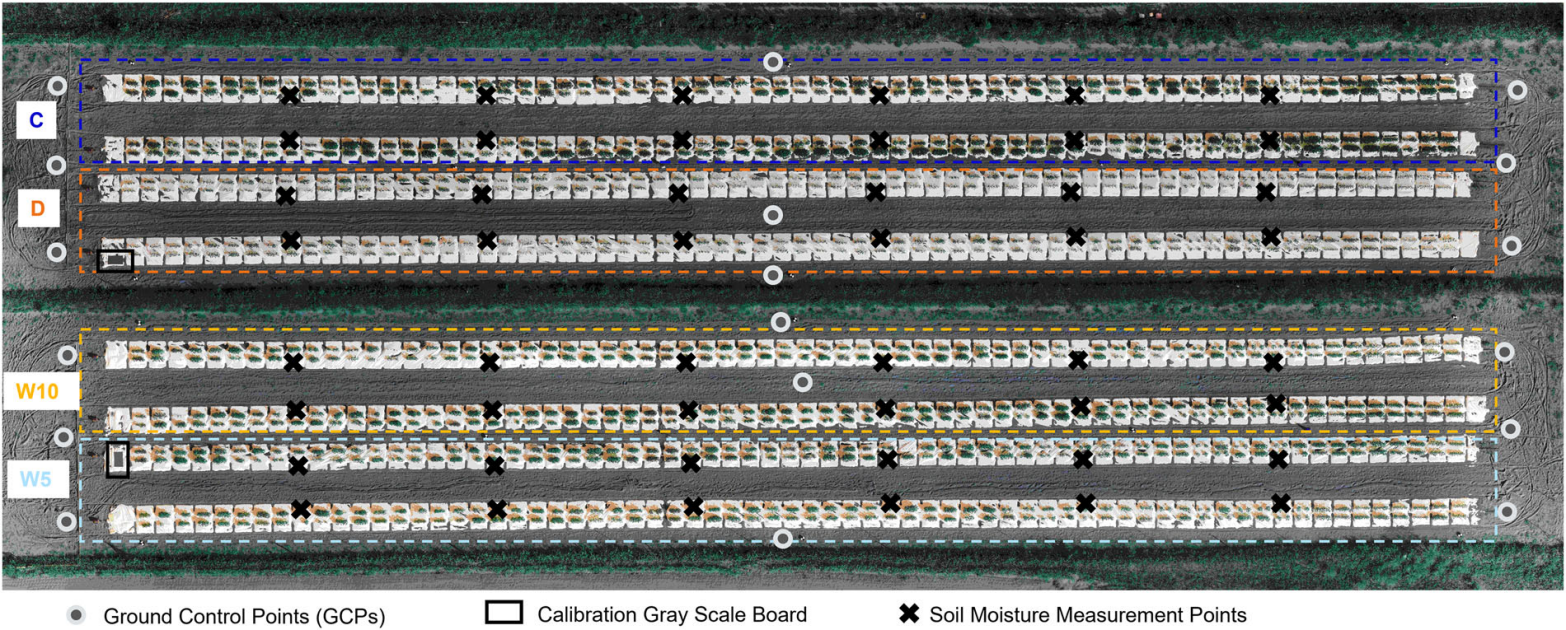
Experimental field overview and treatments: daily watering (control, C), no watering (drought, D), watering for 5 d followed by no watering for 5 d (W5), and watering for 10 d followed by no watering for 10 d (W10)

The diverse panel of soybean accessions used, 198 in total, were obtained from the gene bank of the National Institute of Agrobiological Sciences, Tsukuba, Japan (Supplemental Table S1). These 198 accessions consisted mainly of Japanese and world soybean minicore collections (Kaga et al., 2011; Kajiya‐Kanegae et al., 2021). In 2019, all accessions were cultivated and evaluated at the Arid Land Research Center, Tottori University, Japan (35°32′ N, 134°12′ E; 14 m asl). The experimental field soil was sandy and retained high water permeability. Four phases of watering were employed: daily watering (control, Treatment C), no watering (drought, Treatment D), watering for 5 d followed by no watering for 5 d (Treatment W5), and watering for 10 d followed by no watering for 10 d (Treatment W10). In each of the four treatments, 198 germplasm accessions were placed in two rows with one irrigation tube per row, and microplots were placed in parallel on either side of the irrigation tubes (Figure 1). For each treatment, the 198 accessions were randomly placed. Four plants of each accession were grown in each microplot. The distances between two rows, two microplots, and two plants were 50, 80, and 20 cm, respectively. Fertilizer (13, 6.0, 20, 11, 7.0 g m^−2^ of N, P, K, Mg, and Ca, respectively) was applied to the field prior to sowing. Sowing was performed on 10 July 2019. Two to three seeds were sown at each position, after which, germinated seedlings were thinned to one per position 2 wk after sowing.

**FIGURE 2:**
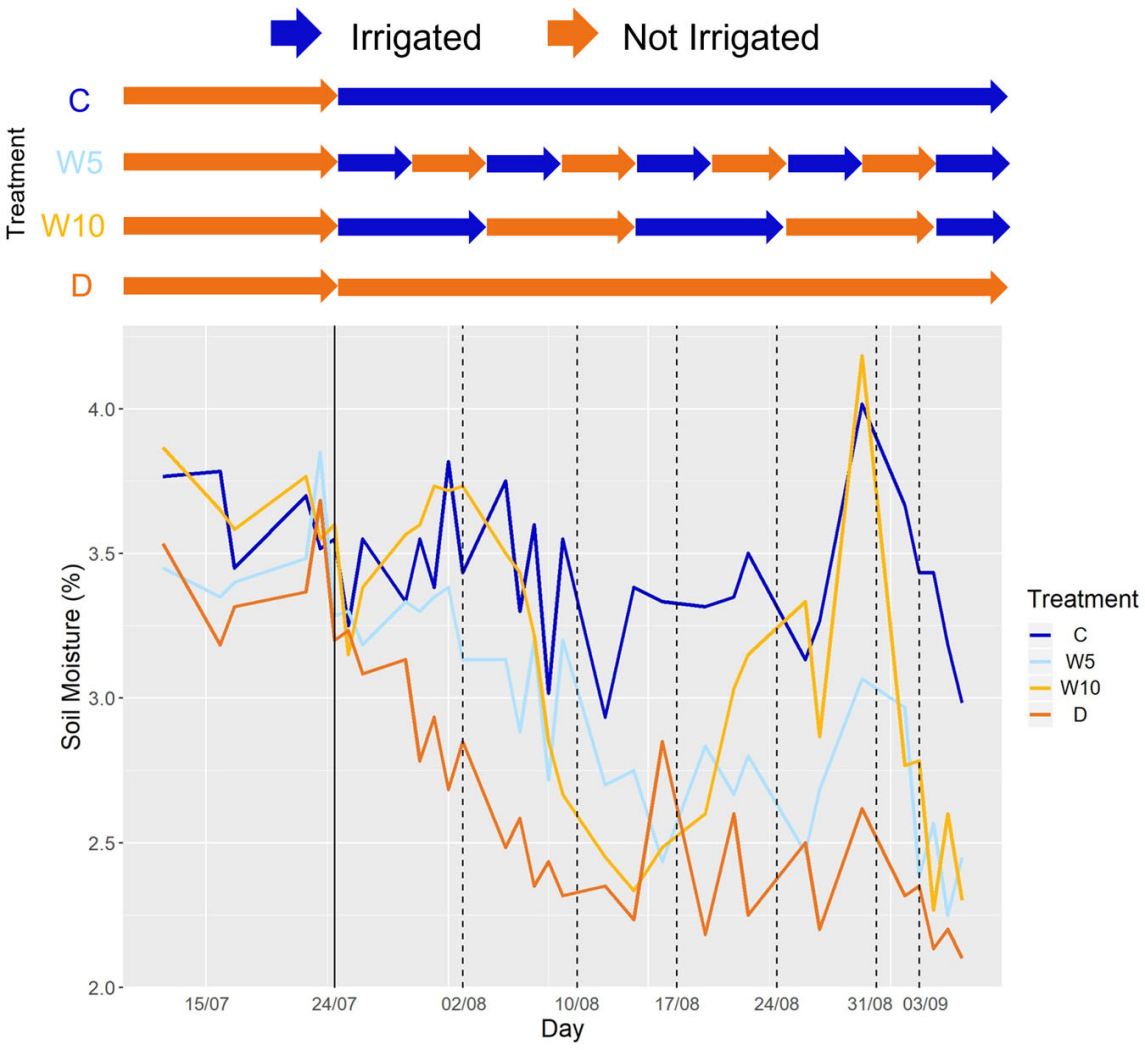
The cycle of irrigation and soil moisture in each treatment: daily watering (control, C), no watering (drought, D), watering for 5 d followed by no watering for 5 d (W5), and watering for 10 d followed by no watering for 10 d (W10). The solid line represents the date of thinning and dashed lines represent the dates for unmanned aerial vehicle (UAV) measurements

White mulching sheets (Tyvek, DuPont) were laid over the ridges to prevent rainwater infiltration into the soil and to control soil drought levels (Figure 1). A watering tube was installed under the mulching sheets at the center of each row. The watering tube (JKC Agro) irrigated at a flow rate of 1.1 L h^−1^ m^−1^. Watering was done over 5 h total daily (7:00‒9:00, 12:00‒14:00, and 16:00‒17:00) starting the day after seedling thinning for Treatments C, W5, and W10. The cycle of irrigation and timing of measurement in each treatment are shown in Figure 2. Soil moisture was measured using a soil moisture meter (TDR‐341F, Fujiwara Seisakusho) at 12 sites for each treatment over 30 d (Figure 1). Except for rainy days, no more than 3 d were allowed between the soil moisture measurements. The soil moisture in each treatment was calculated as the mean of the measurements for each treatment (Figure 2). As shown in Figure 2, because of rainy weather on 28 and 29 August, the soil moisture reached its highest on 30 August in Treatments C and W10 but not in Treatments W5 and D. The effect of measurement location bias may appear because soil moisture was measured from some microplots rather than from all microplots in the field. Some accessions did not germinate, or the plants were too small for MS data collection. Therefore, data from several accessions were not used in the subsequent analyses. The final dataset used contained 190 accessions in Treatment C, 183 in Treatment D, 190 in Treatment W5, and 190 in Treatment W10 (Supplemental Table S1). Days to flowering (DTF), which was defined as the date that 50% of plants flowered in each microplot (Supplemental Figure S1), was measured. To record DTF, we visited each microplot every other day during the flowering period. The shoot dry weight was used as the AGB for each microplot. In each microplot of four plants, two plants located at the center were collected from 9 September to 11 September, and AGB was measured for each plant. The AGB of each microplot was the average of the two measured plants. The ratio of flowering microplots in all treatments to those in each treatment in this trial is summarized in Table 1.

**TABLE 1:** Dates for multispectral (MS) imaging and the destructive measurement. Flowering ratio presents the ratio of flowering microplots over all treatments (All) and in each treatment; daily watering (control), no watering (drought), watering for 5 d followed by no watering for 5 d (W5), and watering for 10 d followed by no watering for 10 d (W10)

			Flowering ratio				
Week	Date	Days after sowing	All	Control	W5	W10	Drought
1	Aug. 2	23	0	0	0	0	0
2	Aug. 10	31	0.1	0.12	0.09	0.1	0.1
3	Aug. 17	38	0.35	0.39	0.33	0.3	0.36
4	Aug. 24	45	0.58	0.61	0.57	0.57	0.59
5	Aug. 31	52	0.77	0.78	0.76	0.75	0.79
6	Sept. 3	55	0.86	0.87	0.85	0.85	0.86
Destructive measurement	Sept. 9	61	0.93	0.94	0.92	0.94	0.93

### 2.2 Multispectral data collection and processing

Multispectral images (1.0 cm pixel^−1^) were collected with a four‐eye MS camera (Xacti) mounted on a quadcopter UAV (DJI Matrice 100, DJI). The MS camera had four independent lenses and sensors attached with different filters (MidOpt) including two triple bandpass filters (TB475/550/850 and TB550/660/850), a red‐edge bandpass (Bi725), and a visible color bandpass (SP644). The MS camera was set for continuous data capture at two frames per second per lens with a total of eight frames per second. The TB475/550/850 can collect spectral intensities at 475 nm (blue), 550 nm (green), and 850 nm (near‐infrared). The TB550/660/850 can collect spectral intensities at 550 nm (green), 660 nm (red), and 850 nm (near‐infrared). The Bi725 can collect a spectral intensity of 725 nm (red‐edge). The SP644 was used to confirm the collected MS images and was not used to extract any spectral information. The spectral intensity of the incident light was measured using an AS7265X Demo Kit (ams‐OSRAM AG) equipped with an 18‐band MS sensor attached to the top of the UAV. We calibrated the MS data based on spectral intensity data from the AS7265X Demo Kit after the MS data were collected. Spectral reflectance was calculated as the ratio of each spectral intensity from a gray‐scale panel (Figure 1). Therefore, this MS camera could collect five types of spectral reflectance: 475 nm (blue), 550 nm (green), 660 nm (red), 725 nm (red‐edge), and 850 nm (near‐infrared). Images were collected six times (once a week) from 2 August to 3 September (Table 1). The UAV flights were set to an altitude of 20 m and scheduled for 12:00 under clear sky conditions daily. The overlap and sidelap rates were set to 90%. Orthomosaic images of four independent lenses were obtained using a Pix4Dmapper (Pix4D, Switzerland). Eighteen ground control points (GCPs) were set up in the field. We measured the positions of these GCPs using Aeropoints (Propeller, NSW, Australia). Using geolocation information from the GCPs, 792 microplots, each with a maximum of four plants, were segmented out of the orthomosaic images. The NDVI‐based segmentation method was used to segment plants from an image of each microplot to extract and analyze the MS image data only from the plants. In the images collected by the UAV, the NDVI differed significantly from the soil surface, white mulching sheets, and plants (Supplemental Figure S2), thus, plants were segmented with the NDVI threshold set to 0.15. These analyses (extraction of spectral reflectance, calculation of NDVI, and NDVI‐based segmentation) were performed using the OpenCV v3.3.1 library in Python v3.6.8.

### 2.3 Vegetation index

Five types of VIs were used: the NDVI (Price & Bausch, 1995; Zarate‐Valdez et al., 2015), green–red vegetation index (GRVI) (Motohka et al., 2010), normalized difference red‐edge index (NDRE) (Jorge et al., 2019), normalized difference index using red‐edge and red spectral reflectance (NDI_red_) (Delegido et al., 2013), and red‐edge triangular vegetation index (RTVI) (Chen et al., 2010). The equations of these VIs are provided in Supplemental Table S2.

The VIs were calculated based on the spectral reflectance values of each plant pixel segmented in each microplot. Although there were four plants in each microplot, MS data were collected as a community of four plants within each microplot without considering the overlap between plants. Although we attempted to segment only the plant pixels, the background noise was not completely removed. In each microplot, the average VI value may have been heavily influenced by background noise. To reduce this effect, the median of all the segmented plant pixels was used as the representative value of each microplot. Calculations of the VIs were performed using R v3.6.0.

### 2.4 Whole‐genome SNP data

Whole‐genome sequence data for all accessions used were obtained (Kajiya‐Kanegae et al., 2021). All accessions were genotyped using the Illumina HiSeq X Ten or HiSeq 4000 (Illumina), and 4,776,813 single‐nucleotide polymorphisms (SNPs) were identified. Among these SNPs, those that were heterozygous or those in which >95% of the individuals had missing data were excluded. The markers were also filtered for a minor allele frequency <0.025, and missing data were imputed based on the mean, after which they were filtered again for a minor allele frequency <0.05. Finally, the linkage disequilibrium was computed only for SNP pairs for which the distance was <25,000 base pairs (bp) and SNPs were selected at linkage disequilibrium below 0.8, resulting in a total of 173,583 SNP markers. This thinning with linkage disequilibrium was performed using the ‘LD.thin’ function in the ’gaston’ package in R v1.5.7 (Perdry & Dandine‐Roulland, 2020). The additive numerator relationship matrix **G** was estimated using the ‘calcGRM’ function in the ‘RAINBOWR’ package in R v0.1.25 (Hamazaki & Iwata, 2020).

### 2.5 Genomic heritability

Genomic heritability (de los Campos et al., 2015) was estimated to determine the level of genetic control of VIs at each growth stage and each treatment. Genomic heritability is the proportion of phenotypic variance explained by genome‐wide SNPs (Litchfield et al., 2015; Stanton‐Geddes et al., 2013). Genomic heritability for each of VIs and for AGB was calculated following **G**:

$$y_{i}=\mu +u_{i}+\varepsilon_{i}$$

where *y_i_* is the phenotypic value for each VI or AGB for genotype *i* (*i* = 1,…, *n*), μ is the overall mean, *u_i_* is the genetic random effect, and ε*_i_* is the residual random effect. Vector $u={\left(u_{1},\text{…}u_{n}\right)}^{T}$ follows the multivariate normal (MVN) distribution, $\mathrm{MVN}(0,G\sigma_{g}^{2})$ where **G** is the additive genomic relationship matrix and $\sigma_{g}^{2}$ is the additive genetic variance. Vector $\varepsilon ={\left(\varepsilon_{1},\text{…}\varepsilon_{n}\right)}^{T}$follows the multivariate normal distribution $\mathrm{MVN}(0,I\sigma_{e}^{2})$, where **I** is the identical matrix and $\sigma_{e}^{2}$ is the residual variance. Genomic heritability was calculated as $h^{2}=\frac{\sigma_{g}^{2}}{\sigma_{g}^{2}+\sigma_{e}^{2}}$. The model was implemented using the ‘EMM.cpp’ function in the ‘RAINBOWR’ package in R v0.1.25 (Hamazaki & Iwata, 2020).

### 2.6 Multitrait model

For each combination of growth stage (week) and treatment, a multitrait (MT) model below was built using a Bayesian multivariate Gaussian model (Lado et al., 2018; Montesinos‐López et al., 2016) to estimate genetic correlation between the AGB and VIs. This model was also used to predict the genotypic values of AGB. In addition, for each treatment, an MT model was built to estimate the genetic correlation between AGB and DTF.

(1) $$\begin{array}{c} \left[\begin{array}{c} y_{0}\\ y_{1}\\ \vdots \\ y_{t} \end{array}\right]=\left[\begin{array}{cccc} X & 0 & \text{…} & 0\\ 0 & X & \ddots & \vdots \\ \vdots & \ddots & \ddots & 0\\ 0 & \text{…} & 0 & X \end{array}\right]\left[\begin{array}{c} b_{0}\\ b_{1}\\ \vdots \\ b_{t} \end{array}\right]+\left[\begin{array}{cccc} Z & 0 & \text{…} & 0\\ 0 & Z & \ddots & \vdots \\ \vdots & \ddots & \ddots & 0\\ 0 & \text{…} & 0 & Z \end{array}\right]\left[\begin{array}{c} u_{0}\\ u_{1}\\ \vdots \\ u_{t} \end{array}\right]+\left[\begin{array}{c} \varepsilon_{0}\\ \varepsilon_{1}\\ \vdots \\ \varepsilon_{t} \end{array}\right] \end{array}$$

where *t* is the number of secondary traits (VIs or DTF), **y**_0_ and **y** *_k_* (*k* = 1, …, *t*) are the vectors of *n* × 1 length phenotypic trait value of AGB and secondary traits, **X** is the fixed effect design matrix (which is the same for AGB and VIs), **b** *_k_* (*k* = 0, …, *t*) is the vector of fixed effects of flowering (i.e., before or after flowering based on DTF on each measurement day) for trait *t*. **Z** is the random effect design matrix (the same for all traits), **u** *_k_* is the vector of genetic random effects of trait *k*, and **ε** *_k_* is the vector of residual random effects of trait *k*. The vector $\left[\begin{array}{c} u_{0}\\ \vdots \\ u_{t} \end{array}\right]$ follows a multivariate normal distribution MVN(0,G⊗Σ), where **G** is the additive genomic relationship matrix and **Σ** is the genetic variance–covariance matrix across traits. The vector $\left[\begin{array}{c} \varepsilon_{0}\\ \vdots \\ \varepsilon_{t} \end{array}\right]$ follows the multivariate normal distribution MVN(0,I⊗R), where **I** is the identical matrix, **R** the residual variance–covariance matrix across traits, and **I** the identical matrix. Matrices **Σ** and **R** were estimated as a *t* × *t* unstructured matrix. To predict the genotypic values of AGB for each treatment, we built two types of MT models: (a) with the five types of VIs (MT_all_ model) and (b) with only NDVI (MT_NDVI_ model). When the MT_all_ model is more accurate than the MT_NDVI_ model, it is useful to collect not only NDVI, but also the other four types of VIs. This model was built using the ‘MTM’ R package v1.0.0 (de los Campos, 2020).

### 2.7 Kernels based on relationship matrices

The VI relationship model for each treatment (**V**_each_) was calculated for each combination of a growth stage (week) and a treatment; **V**_each_ was calculated as $S_{\mathrm{each}}S_{\mathrm{each}}^{T}$, where **S**_each_ is a *n* × *t* matrix of scaled phenotypic values of VIs (*t* = 5) for each genotype. The VI relationship model over all treatments (**V**_all_) was calculated for each growth stage over all treatments; **V**_all_ was calculated as $S_{\mathrm{all}}S_{\mathrm{all}}^{T}$, where **S**_all_ is a nm × *t* matrix of scaled phenotypic values of VIs for each microplot, where nm indicates the number of microplots over all treatments. A kernel based on the field heterogeneity relationship matrix (**F**) was defined based on the Euclidean distances between microplots based on the positions in the field. Plots belonging to different rows were assumed to be independent; thus, the Euclidean distance was set to infinity in this case. The Euclidean distances were converted to a Gaussian kernel, where the (*i*, *j*)th element of said kernel is defined as follows:

$$f_{ij}=\;\exp\left(-hd_{ij}^{2}\right)$$

where $d_{ij}^{2}$ is the squared distance between microplots, and *h* is the reciprocal of the median of squared distances between all combinations of all microplots. The kernel representing the covariance structure in the genotype × environment interaction (GEI) effect (**GE**) was defined for all treatments; **GE** was calculated as$\;\left(Z_{g}GZ_{g}^{T}\right)\circ \left(Z_{E}Z_{E}^{T}\right)$, where **Z_g_** and **Z_E_** are incidence matrices for genotypes and treatments, respectively (Jarquín et al., 2014; Pérez‐Rodríguez et al., 2015, 2017).

### 2.8 Single‐ and multiple‐kernel models within each treatment

To predict the phenotypic values of AGB for each treatment, we built four kernel regression models. In addition, we built a simple linear regression model to predict AGB using NDVI as an input variable. All built models are summarized in Table 2.

**TABLE 2:** Prediction models within each treatment

Model name	Kernel^a^	Equation^b^	Index
*G* model	**G**	$y_{il}=b_{l}+u_{i}+\varepsilon_{i}$	Equation S1
*V*_each_ model	**V**_each_	$y_{il}=b_{l}+v_{i}+\varepsilon_{i}$	Equation S2
*G* + *V*_each_ model	**G**, **V**_each_	$y_{il}=b_{l}+u_{i}+v_{i}+\varepsilon_{i}$	Equation S3
*G* + Field model	**G**, **F**	$y_{il}=b_{l}+u_{i}+f_{i}+\varepsilon_{i}$	Equation S4
NDVI_each_ model	–	$y_{il}=b_{l}+a{\mathrm{NDVI}}_{i}+\varepsilon_{i}$	–

^a^ **G**, the additive genomic relationship kernel; **V**_each_, the vegetation indices (VIs) relationship kernel for each treatment; **F**, the field heterogeneity relationship kernel.

^b^ *y_il_*, the phenotypic value of aboveground biomass (AGB) for genotype *i* (*i* = 1, …, *n*); *b_l_*, the fixed effect of flowering (i.e., before or after flowering based on days to flowering on each measurement day) (*l* = 1, 2); *u_i_*, the genetic random effect; *v_i_*, the random effect of the VIs; *f_i_*, the random effect reflecting the among‐plot distances; ε*_i_*, the residual random effect; NDVI*_i_*, the scaled observed value of normalized difference vegetation index (NDVI); *a*, the regression coefficient of NDVI*_i_*.

The single‐kernel model with **G** (*G* model) compares the utility of a VI‐based model to a standard genome‐wide marker‐based model. The single‐kernel model with **V**_each_ (*V*_each_ model) evaluates the usefulness of VIs in predicting the phenotypic values of AGB within each irrigation level. This model can be used to predict the phenotypic values of AGB based on MS image data at a low cost without genome‐wide marker data and destructive sampling. The multikernel model with **G** and **V**_each_ (*G* + *V*_each_ model) was employed to compare whether the combination of genome‐wide marker and VIs data would increase the prediction accuracy. The multikernel model with **G** and **F** (*G* + Field model) aimed to confirm whether the field heterogeneity affected the phenotypic values of AGB within each treatment. When this model exhibits no or little increment of the prediction accuracy compared with the **G** single‐kernel model, then the location effect of the microplots (i.e., distance dependent among‐plot heterogeneity) can be assumed to be small in this experimental field. The simple linear regression model with NDVI (*NDVI*_each_ model) aimed to validate the usefulness of the four VIs with NDVI excluded.

The details of these four types of single‐ and multiple‐kernel models are described in Supplemental File S1.

### 2.9 Single‐ and multiple‐kernel models over all treatments

To evaluate whether MS imaging can capture the variability in AGB caused by differences in irrigation levels, we built models using data from all treatments. If MS data could capture the variability in AGB, we could predict the phenotypic value of AGB for untested genotypes based on MS data. We built five kernel regression models. In addition, we built a simple linear regression model to predict AGB using NDVI as an input variable. All built models are summarized in Table 3.

**TABLE 3:** Single‐ and multiple‐kernel models over all treatments

Model name	Kernel^a^	Equation^b^	Index
*G + E* model	**G**	$y_{ijl}=E_{j}+b_{l}+u_{i}+\varepsilon_{ij}$	Equation S5
*G + E +* GEI model	**G**, **GE**	$y_{ijl}=E_{j}+b_{l}+u_{i}+{\mathrm{ge}}_{ij}+\varepsilon_{ij}$	Equation S6
*G + E +V*_all_ model	**G**, **V**_all_	$y_{ijl}=E_{j}+b_{l}+u_{i}+v_{ij}+\varepsilon_{ij}$	Equation S7
*G + V*_all_ model	**G**, **V**_all_	$y_{ijl}=b_{l}+u_{i}+v_{ij}+\varepsilon_{ij}$	Equation S8
*V*_all_ model	**V**_all_	$y_{ijl}=b_{l}+v_{ij}+\varepsilon_{ij}$	Equation S9
NDVI_all_ model	–	$y_{ijl}=b_{l}+a{\mathrm{NDVI}}_{ij}+\varepsilon_{ij}$	–

^a^ **G**, the additive genomic relationship kernel; **GE**, genotype × environment interaction kernel; **V**_all_: the vegetation indices relationship kernel over all treatments.

^b^ *y_ijl_*, the phenotypic value of aboveground biomass (AGB) for genotype *i* (*i* = 1, …, *n*) in treatment *j* (*j* = 1, …, 4); *E_j_*, the fixed effect for treatment; *b_l_*, the fixed effect of flowering (i.e., before or after flowering based on days to flowering on each measurement day) (*l* = 1, 2); *u_i_*, the genetic random effect; ge*_ij_*: the genotype × environment interaction random effect; *v_il_*, the random effect of the VIs; ε*_ij_*, the residual random effect; NDVI*_ij_*, the scaled observed value of normalized difference vegetation index (NDVI); *a*, the regression coefficient of NDVI*_ij_*.

The aim of each of the six models is as follows. The single‐kernel model with **G** and *E* (*G + E* model) aimed to compare the utility of a VI‐based model to a standard genome‐wide marker data and treatment information‐based model. A multikernel model with **G**, *E*, and GEI (*G + E +* GEI model) was used to confirm whether GEI affected the phenotypic values of AGB over all treatments. When little or no increase in the prediction accuracy is exhibited by *G + E +* GEI model vs. the *G + E* model, the effect of GEI can be assumed to be small in this experimental field. The multikernel model with **G**, *E*, and **V**_all_ (*G + E +V*_all_ model) aimed to evaluate the usefulness of VIs in modeling the GEI. When this model outperformed the prediction accuracy of the **G**, *E*, and GEI multikernel model, then the MS image data may be a useful phenotype for modeling GEI. The multikernel model with **G** and **V**_all_ (*G + V*_all_ model) was aimed to evaluate whether the differences in irrigation levels are reflected on VIs. This model can be used in fields in which irrigation levels are largely complex and uneven. The single‐kernel model with **V**_all_ (*V*_all_ model) was aimed to evaluate the usefulness of VIs for predicting the phenotypic values of AGB over all irrigation levels. This model can be used for predicting phenotypic values of AGB based on MS image data at a low cost (without genome‐wide marker data and destructive sampling). The simple linear regression model with NDVI (NDVI_all_ model) aimed to validate the usefulness of using four VIs with NDVI excluded.

The details of these five single‐ and multiple‐kernel models are described in Supplemental File S1. All single‐ and multiple‐kernel models were built using ‘EM3.cpp’ function in the ‘RAINBOWR’ package in R v0.1.25 (Hamazaki & Iwata, 2020).

### 2.10 Evaluation of prediction accuracy

The prediction accuracy of each model was evaluated using fivefold cross‐validations with 10 replications. The accuracy of the multitrait model was evaluated by using AGB only in the training set, whereas the phenotypic data from the VIs were used in both the training and test sets. In the single‐ and multiple‐kernel models for all treatments, the genotypes used as training data were not used for the test data. The distribution of AGB values in each treatment did not follow a normal distribution, thus, the genomic heritability of AGB was calculated after taking the logarithm, ensuring that the error term follows a normal distribution. The MT and single‐ and multiple‐kernel models were built using these log values for AGB. Pearson correlations were calculated between the observed log AGB values and the predicted values.

## 3 RESULTS

### 3.1 Effects of treatments, accessions, and DTF on AGB

To evaluate the significance of the main effects of treatments and accessions on AGB, analysis of variance (ANOVA) was performed on AGB with two plant replications in each microplot. The ANOVA indicated significant differences (*p* < .001) between treatments and accessions for AGB (Supplemental Table S3). Next, we examined the effect of flowering on AGB. Because DTF was measured on a microplot basis and there was no plant replication in each microplot, the mean AGB in each microplot was used in the analysis. Genetic correlations between DTF and AGB were 0.33, 0.18, 0.15, and −0.07 in Treatments C, W5, W10, and D, respectively. In addition, the ANOVA test showed significant effects of flowering (i.e., before or after flowering on each measurement day based on DTF) on AGB only in Treatment C from Weeks 2 and 4 (*p* < .001) (Supplemental Table S4).

### 3.2 Heritability and relationship among VIs

**FIGURE 3:**
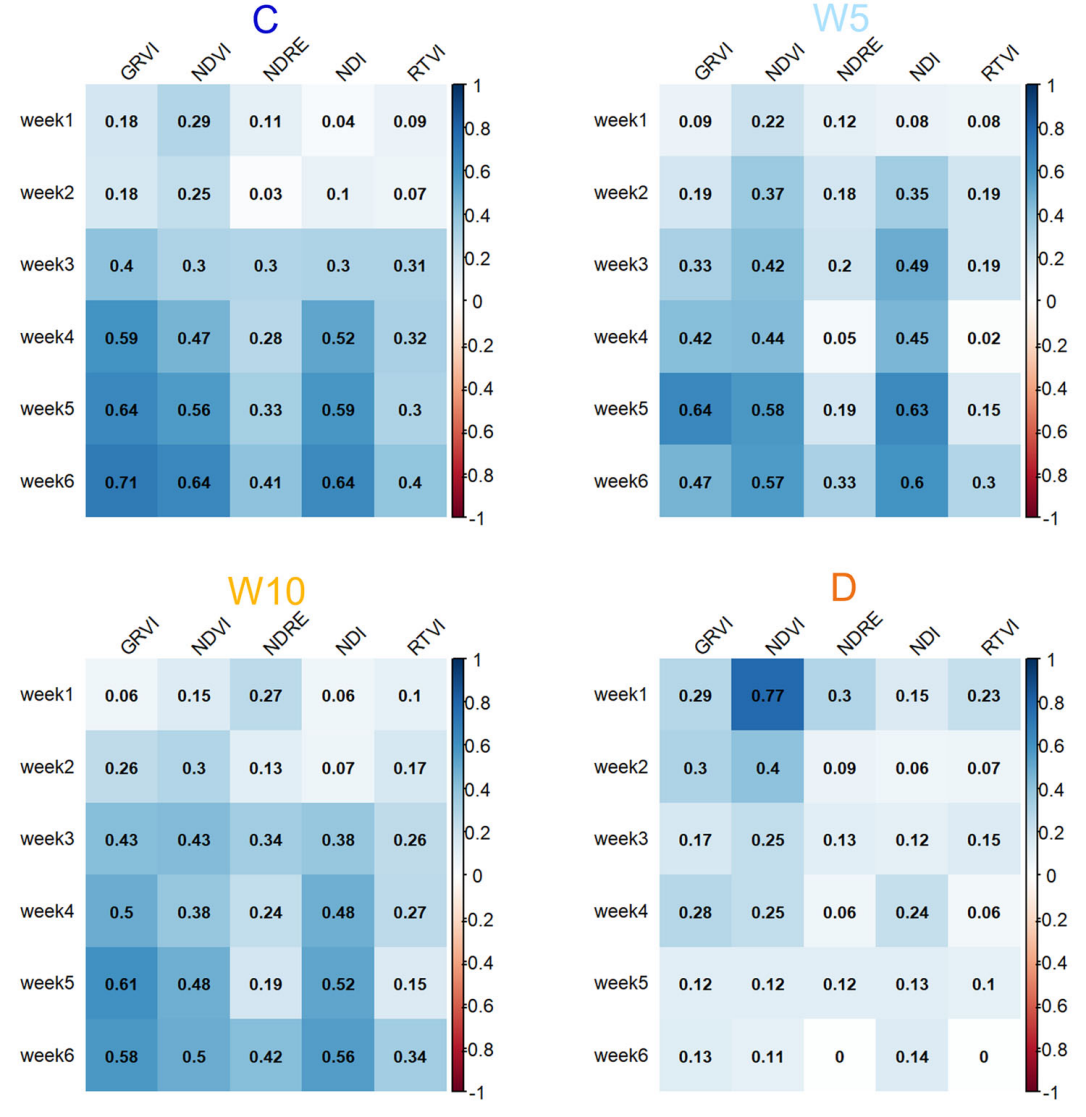
Genomic heritability for the vegetation indices in each treatment: daily watering (control, C), no watering (drought, D), watering for 5 d followed by no watering for 5 d (W5), and watering for 10 d followed by no watering for 10 d (W10). GRVI, green–red vegetation index; NDVI, normalized difference vegetation index; NDRE, normalized difference red‐edge index; NDI, normalized difference index; RTVI, red‐edge triangular vegetation index

The average AGB decreased with decreasing irrigation level (Table 4). Similarly, the genomic heritability of AGB was the highest in Treatment C and lowest in Treatment D. This trend was also observed for the VIs (Figure 3). For VIs, we evaluated the genomic heritability not only for the different irrigation levels but also for the different growth stages. The genomic heritability of the VIs increased in the later growth stages with the exception of Treatment D. The results suggested that AGB and VIs under severe drought conditions were more strongly affected by nongenetic sources of variation than under better‐irrigated conditions. Principal component analysis (PCA) of five VIs was performed for each treatment and across all irrigation levels for the different growth stages. The PCA results indicated that the proportion of total variance explained by the first principal component (PC1) increased over time in all treatments (Table 5). In Week 5, in Treatments C, W5, and W10, over 83% of the total variance was explained by PC1. However, in Treatment D, only 73% of the total variance was explained by PC1. The PCA results of all treatments were shown in Supplemental Table S5.

**TABLE 4:** Mean aboveground biomass (AGB) with standard error (SE) and genomic heritability in each treatment; daily watering (control), no watering (drought), watering for 5 d followed by no watering for 5 d (W5), and watering for 10 d followed by no watering for 10 d (W10)

Treatment	Control	W5	W10	Drought
Mean (g) ± SE	49.06 ± 1.48	41.88 ± 1.06	35.68 ± 0.91	11.32 ± 0.36
Genomic heritability	.37	.31	.34	.20

**TABLE 5:** The cumulative proportion of principal component (PC) analysis for five vegetation indices (VIs) in each treatment: daily watering (control, C), no watering (drought, D), watering for 5 d followed by no watering for 5 d (W5), and watering for 10 d followed by no watering for 10 d (W10) for the different growth stages

Week	Treatment	PC1	PC2	PC3	PC4	PC5
1	Control	0.52	0.9	0.98	0.99	1
	W5	0.53	0.89	0.98	0.99	1
	W10	0.53	0.92	0.98	0.99	1
	Drought	0.58	0.9	0.98	0.99	1
2	Control	0.51	0.94	0.99	1	1
	W5	0.53	0.92	0.99	1	1
	W10	0.5	0.91	0.98	0.99	1
	Drought	0.56	0.87	0.99	0.99	1
3	Control	0.82	0.97	1	1	1
	W5	0.75	0.96	0.99	1	1
	W10	0.77	0.95	0.99	1	1
	Drought	0.56	0.89	0.99	1	1
4	Control	0.77	0.97	1	1	1
	W5	0.75	0.97	1	1	1
	W10	0.84	0.97	0.99	1	1
	Drought	0.56	0.93	0.99	1	1
5	Control	0.85	0.97	1	1	1
	W5	0.85	0.99	1	1	1
	W10	0.83	0.98	1	1	1
	Drought	0.73	0.95	1	1	1
6	Control	0.85	0.97	0.99	1	1
	W5	0.84	0.98	1	1	1
	W10	0.84	0.97	1	1	1
	Drought	0.8	0.96	1	1	1

### 3.3 Phenotypic and genetic correlation of AGB and VIs across four drought treatment levels

**FIGURE 4:**
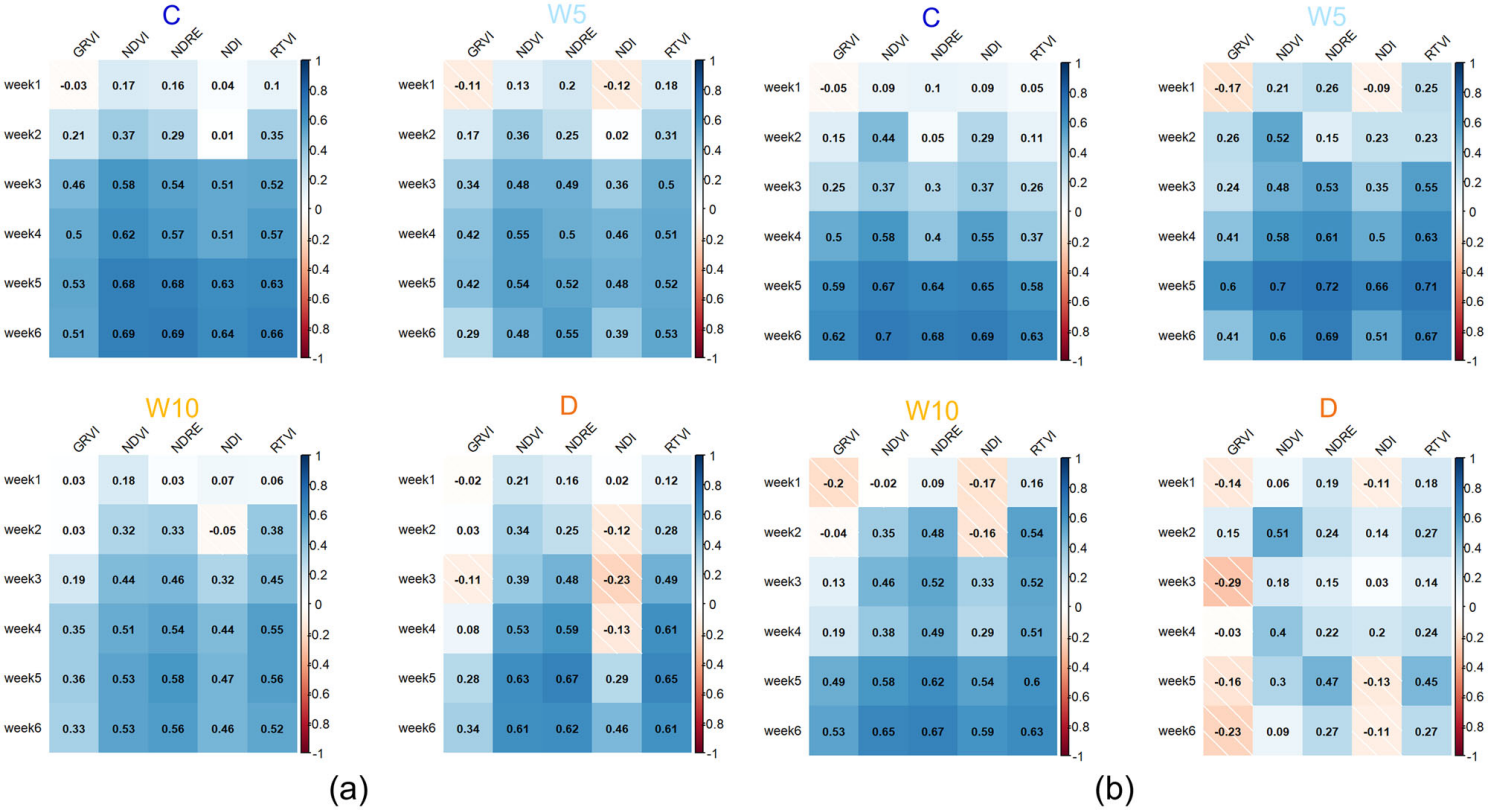
Phenotypic and genetic correlations between aboveground biomass (AGB) and the vegetation indices (VIs) in each treatment: daily watering (control, C), no watering (drought, D), watering for 5 d followed by no watering for 5 d (W5), and watering for 10 d followed by no watering for 10 d (W10). (a) Phenotypic correlation between AGB and VIs in each treatment. (b) Genetic correlation between AGB and VIs in each treatment. GRVI, green–red vegetation index; NDVI, normalized difference vegetation index; NDRE, normalized difference red‐edge index; NDI, normalized difference index; RTVI, red‐edge triangular vegetation index

We focused on the phenotypic correlation between AGB on one hand and VIs on the other. The correlation between AGB and each individual VI was similar among the different irrigation levels (Figure 4a). Three VIs (NDVI, NDRE, and RTVI) were positively correlated with AGB across all treatments. However, in Treatment D, the sign of the correlation between NDI and AGB was initially negative but became positive from Week 5. Genetic correlation was derived from the among‐trait genetic variance–covariance matrix (**Σ**) estimated following the MT model (Equation 1). In the Week 1, the highest genetic correlation between AGB and VIs under each treatment was low: Treatment C, 0.09; W5, 0.26; W10, −0.17; and D, 0.19 (Figure 4b). In Week 6, the genetic correlations increased to 0.70, 0.72, and 0.67 for Treatments C, W5, and W10, respectively. Thus, Treatments C, W5, and W10 showed high genetic correlation, whereas in Treatment D, the highest genetic correlation between AGB and VI only increased to 0.51. The genetic correlation between VIs and AGB in Treatments C, W5, and W10 increased drastically from Weeks 4 and 5, with an average increase of 43%. The flowering ratios based on DTF over all treatments were 0.58 and 0.77 in Week 4 and Week 5, respectively (Table 1). Overall, these results indicate that genetic correlations between VIs and AGB varied during growth and between irrigation levels.

### 3.4 Prediction accuracy of AGB using MT model

**FIGURE 5:**
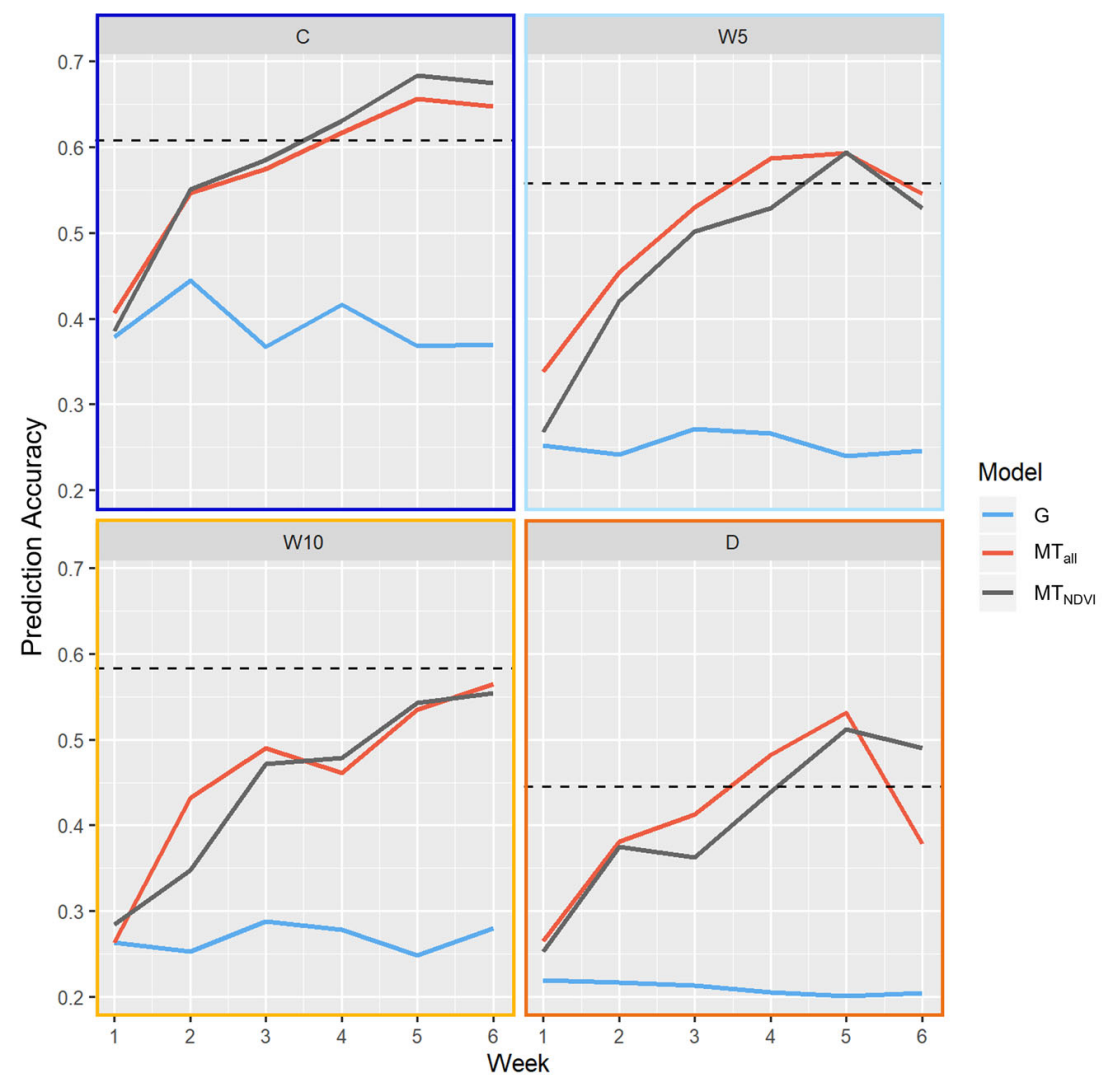
Aboveground biomass (AGB) prediction accuracies from additive genomic relationship kernel model (*G* model), multitrait (MT) model with the five types of vegetation indices (VIs) (MT_all_ model), and with only normalized difference vegetation index (NDVI) (MT_NDVI_) in each treatment: daily watering (control, C), no watering (drought, D), watering for 5 d followed by no watering for 5 d (W5), and watering for 10 d followed by no watering for 10 d (W10). Prediction accuracies were estimated as the correlation between observed log values and predicted values. Dashed lines show the upper limit of prediction accuracy based on the genomic heritability of AGB in each treatment

The prediction accuracy of the genotypic values for AGB with the *G*, MT_all_, and MT_NDVI_ models was evaluated using fivefold cross‐validation (Figure 5). With flowering as the fixed effect, the prediction accuracy of the *G* model was improved by an average of 3% in Treatment C, whereas, on average, the accuracy was not improved in Treatments W5 and W10. With flowering as the fixed effect, the prediction accuracy of the *G* model deteriorated by an average of 5% in Treatment D (Supplemental Figure S3). First, we compared the prediction accuracy of the *G* model and the two types of MT models. The mean accuracy of the *G* model for Treatments C, W5, W10, and D was 0.39, 0.25, 0.27, and 0.21, respectively. Both of the MT models were able to predict AGB more accurately than the *G* model for all irrigation levels. It is evident that in Weeks 5 and 6, some of the MT model prediction accuracies exceed the dashed lines, which indicate the expected prediction accuracy based on the genomic heritability of AGB in each treatment. This may be because the MS data partly explained the variation of AGB that was not from genetic variation. Next, we compared the prediction accuracies of the MT_all_ and MT_NDVI_ models. The prediction accuracy of the MT_all_ model outperformed that of the MT_NDVI_ model by 7%, on average, in Treatment W5, whereas the difference was not large (<3%) in the other treatments.

### 3.5 Prediction accuracy of AGB with single‐ and multiple‐kernel regression models

**FIGURE 6:**
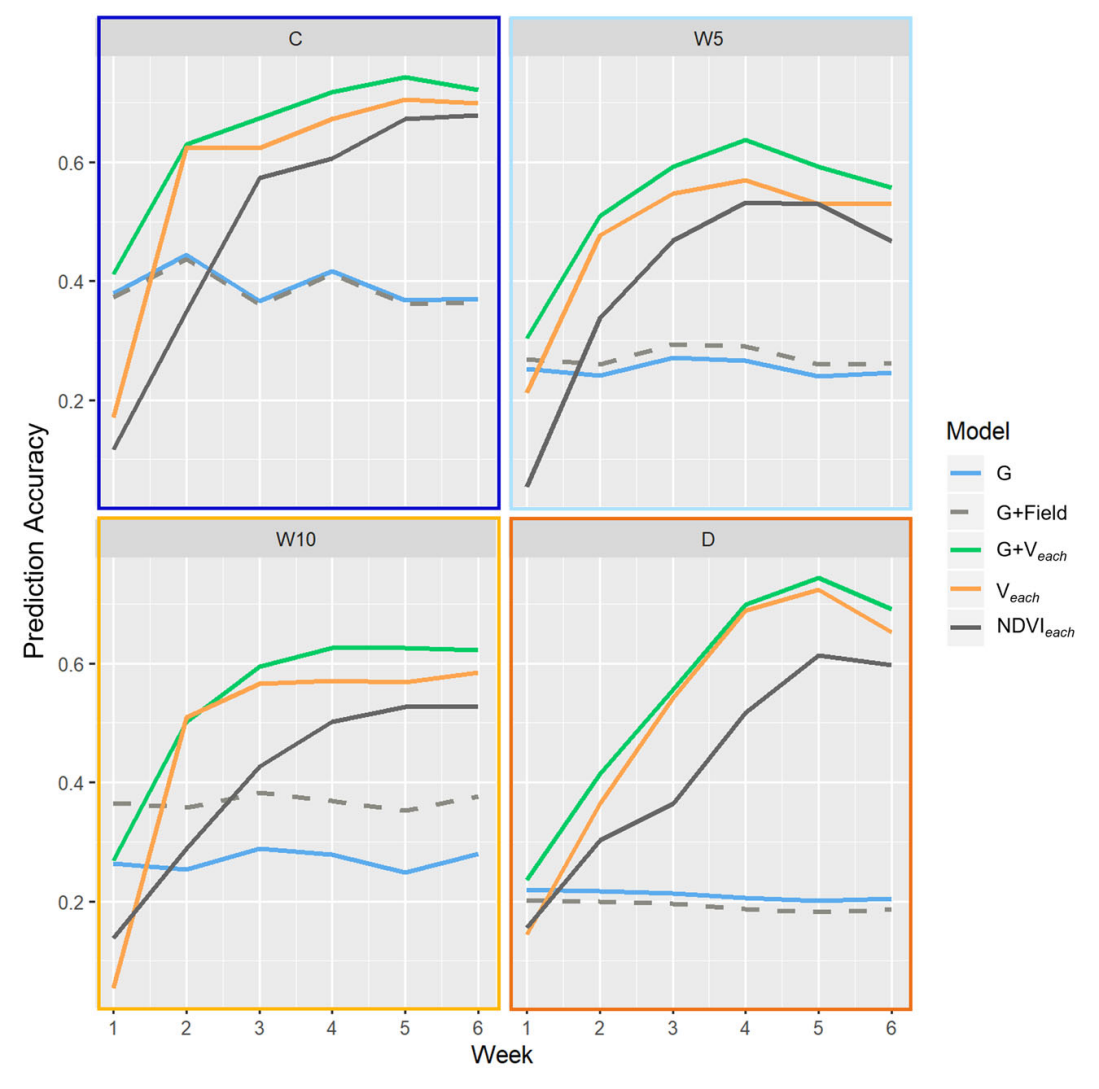
Aboveground biomass (AGB) prediction accuracies from four types of single‐ and multiple‐kernel regression models and a simple linear regression model using normalized difference vegetation index (NDVI) as an input variable in each treatment: daily watering (control, C), no watering (drought, D), watering for 5 d followed by no watering for 5 d (W5), and watering for 10 d followed by no watering for 10 d (W10). The prediction accuracy was estimated as the correlation between observed log values and predicted values. G, the additive genomic relationship model; V_each_, the vegetation indices (VIs) relationship model for each treatment; Field, the field heterogeneity relationship model; NDVI_each_, simple linear regression model with normalized difference vegetation index

Except for Week 1, the prediction accuracy of model *V*_each_ was higher than that of model *G* for all treatments (Figure 6). In Week 2, the prediction accuracy of model *V*_each_ was 77% higher than that of model *G* for all treatments. The change in the prediction accuracy of model *V*_each_ over time showed a different trend between Treatment D and other treatments. In Treatments C, W5, and W10, the maximum value of prediction accuracy in model *V*_each_ and the prediction accuracy in model *V*_each_ of Week 2 changed by only 13, 19, and 15%, respectively. In Treatment D, the maximum value of the prediction accuracy of model *V*_each_ and the prediction accuracy of model *V*_each_ for Week 2 changed by 99%. The prediction accuracy of Treatment D gradually increased over time. Except for Week 1, the prediction accuracy of model *V*_each_ was higher than that of model NDVI_each_ for all the treatments. Particularly in Treatments C, W5, and W10, model *V*_each_ outperformed model NDVI_each_ by 79, 41, and 76%, respectively, in prediction accuracy in Week 2. In Treatment D, *V*_each_ outperformed the model NDVI_each_ by only 20% in terms of prediction accuracy in Week 2. In Week 1, the prediction accuracy of model *G* + *V*_each_ was higher than that of model *V*_each_ by 160%, on average, for all treatments, but from Week 2 through Week 6, the difference in prediction accuracy was not so large. Except for Treatment W10, model *G* + Field barely increased the prediction accuracy of model *G*.

### 3.6 Prediction accuracy for AGB across all drought treatment conditions

**FIGURE 7:**
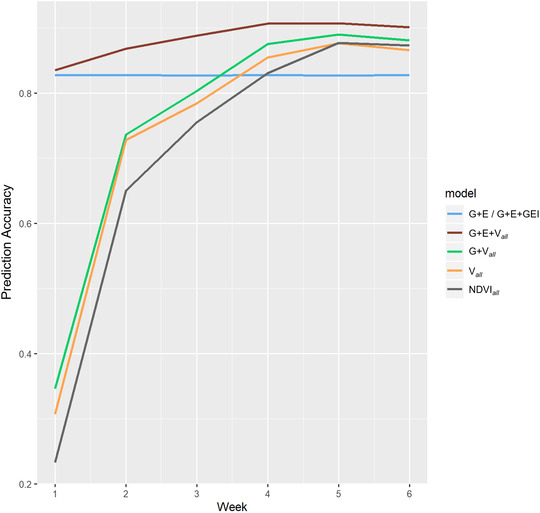
Aboveground biomass (AGB) prediction accuracies from five types of single and multiple kernel regression models and a simple linear regression model using normalized difference vegetation index (NDVI) as an input variable across all treatments. Prediction accuracy was estimated as the correlation between observed log values and predicted values. G, the additive genomic relationship model; GEI, genotype × environment interaction model; V_all_, the vegetation indices relationship model over all treatments; NDVI_all_, simple linear regression model with normalized difference vegetation index

Models *G* + *V*_all_ and *V*_all_ outperformed models *G* + *E* and *G* + *E* + GEI from Week 4 through Week 6 (Figure 7). These results suggest that the MS image data captured the variance in AGB in plants that was not captured by the differences in the irrigation level or genomic information. In addition, there was no difference between the prediction accuracies of models *G* + *E* and *G* + *E* + GEI. The prediction accuracy of model *G* + *E* and *G* + *E* + *V*_all_ was always higher (by 7%, on average) than that of model *G* + *E* and *G* + *E* + GEI. Therefore, the MS image data captured the variation in AGB, which was not from GEI. The prediction accuracies of the three models (*G* + *E* + *V*_all_, *G* + *V*_all_, and *V*_all_) using MS image data increased from Week 1 through Week 4, after which they did not change much, as most of the phenotypic correlations between AGB and each VI plateaued in Week 4 (Supplemental Figure S4). Next, the prediction accuracy of model *V*_all_ was higher (by 13%, on average) than that of model NDVI_all_ from Week 1 through Week 4. However, the prediction accuracy of models *V*_all_ and NDVI_all_ was not very different (>1%) from that of Weeks 5 and 6.

## 4 DISCUSSION

### 4.1 Heritability and PCA of VIs

Overall, we found that the genomic heritability of VIs increased with the progression of growth stage (Figure 3). This is consistent with previous work (Parmley et al., 2019) reporting that the genomic heritability of VIs was higher in the later growth stage in soybean. This result indicated that genetic variation in the MS image data was smaller in the early growth stage than in the late growth stage. However, in our Treatment D, heritability of the VIs did not exhibit this increase. Therefore, in Treatment D, nongenetic variance largely affected the MS data. One reason for this could be the small size of the sampled plants. Because the plant size in Treatment D was significantly smaller than that in the other treatments, the number of pixels that belonged to the plants was small (Supplemental Figure S5). When the plants are small, reflectance from the ground can affect the MS data acquired (Christenson et al., 2014; Cure et al., 1989).

The PCA of the five types of VIs showed that the proportion of variation explained by the first two components was 95% after Week 5 (52 d after sowing) in all treatments. Accumulated variance in the first two principal components of the data, which consisted of six types of VIs, including NDVI, obtained at the R5 stage (80 d after sowing) of soybean, was reported as 95.39% (da Silva Junior et al., 2018). The results of the present study are consistent with those of the previous study (da Silva Junior et al., 2018). Additionally, in the present study, the proportion of variance explained by PC1 increased over time. This result suggests the importance of obtaining multiple VIs before the flowering stage.

### 4.2 Phenotypic and genetic correlations

The correlation between AGB and VIs was lower in Treatments W5 and W10 than in Treatments C and D during the late growth stages (Figure 4a). Treatments W5 and W10 were not irrigated at Week 5 and Week 6. These treatments were irrigated from 4 September (after Week 6). This change in irrigation (i.e., irrigated or not irrigated) might be a confounding factor, and the correlation between AGB and VIs was not as high in Treatments W5 and W10 as in Treatments C and D. In Treatment D, the correlation between NDI and AGB showed a negative correlation at first and gradually changed to a positive correlation over time. The NDI has been reported to strongly correlate with the leaf area index in various crops including soybean (Delegido et al., 2011, 2013). Therefore, genotypes that had a large leaf area index during Weeks 1 through 4 finally might not withstand drought stress and show low AGB.

Genetic correlation between AGB and the VIs, as estimated using the MT model, showed a clear difference between Treatment D and the other treatments (Figure 4b). In Treatments C, W5, and W10, the genetic correlation between AGB and the VIs increased to 0.7, 0.72, and 0.67, whereas it increased up to 0.51 in Treatment D. This suggests that the high phenotypic correlation mainly is due to the high genetic correlation under irrigated conditions, whereas the high phenotypic correlation is due to the high residual correlation under drought conditions. Therefore, it is suggested that VIs can capture AGB variability regardless of the source of variation (genetic or nongenetic).

### 4.3 MT model prediction

The prediction accuracy for the genotypic values of AGB was improved in the MT_all_ and MT_NDVI_ models compared with that in the *G* model. To predict a target trait with low heritability, such as yield or biomass, it would be useful to incorporate secondary traits that have high heritability and genetic correlation with a target trait (Bhatta et al., 2020; Jia & Jannink, 2012).

In this study, there was no significant difference between the prediction accuracies of the MT_all_ and MT_NDVI_ models for all treatments. Studies have also reported that the accuracy of the MT model did not increase significantly with an increase in the number of secondary traits (Bao et al., 2015; Schulthess et al., 2016). Furthermore, the inclusion of two vegetation indices with low heritability (NDRE and RTVI) in the MT model, such as Week 6 in Treatment D, may have reduced the prediction accuracy of the MT_all_ model compared with that of the MT_NDVI_ model. In contrast, in Treatment W5, the prediction accuracy of the MT_all_ model was higher than that of the MT_NDVI_ model in Weeks 1 through 4. The prediction accuracy of the MT model is reported to be improved by increasing the number of secondary traits (Wang et al., 2017). This difference may be caused by the heritability of the secondary traits. Therefore, secondary traits with low heritability should not be incorporated into the MT model, although increasing the number of secondary traits may increase prediction accuracy.

Spectral reflectance in soybean is influenced by plant maturity (Zhang et al., 2019). Spectral reflectance measurements have also been reported to capture physiological parameters associated with relative maturity in wheat (Krause et al., 2019). Therefore, wheat grain yield correction based on the variations in days to heading reduced the advantage of the MT model using NDVI as a secondary trait over the *G* model (Rutkoski et al., 2016). In the present study, the factors of flowering and nonflowering were included in the models as fixed effects; however, the advantage of the MT model over the *G* model was not reduced in soybean. One reason for this may be the low genetic correlation between AGB and DTF. The genetic correlation between AGB and days to heading was estimated at −0.71 in severe drought treatment by Rutkoski et al. (2016) for wheat. In the present study, the genetic correlation between AGB and DTF was estimated to be −0.07 for soybean. In addition, vegetative growth and reproductive growth switch after ear heading in wheat (Slafer & Rawson, 1994), whereas in soybean, vegetative growth continues to some extent after flowering (Fehr & Caviness, 1977). These differences may explain why the advantage of the MT model over the *G* model was not reduced in soybean.

The prediction accuracy of the MT_all_ model outperformed that of the *G* model by Week 2. In Week 2, the percentage of microplots that flowered in all treatments was only 10% (Table 1). Therefore, the MS data collected before flowering can be used to predict future genotypic values. This may enable us to determine cross‐combinations before flowering based on the predicted values. Because the prediction accuracy of the MT_all_ model was higher than that of the *G* model, which used genomic information alone, breeding may be more efficiently performed by collecting MS data before flowering.

### 4.4 Single‐ and multiple‐kernel regression models

In the single‐ and multiple‐kernel prediction, models *G* + *V*_each_ and *V*_each_ outperformed model *G* in all treatments except in Week 1 (Figure 6). The models using kernel **V**_each_ increased the prediction accuracy of AGB compared with model *G* because they could leverage the phenotypic correlations between the VIs and AGB. In Treatments C, W5, and W10, there was no significant increase from Week 2 (almost before flowering) to Week 5 (almost after flowering) in the prediction accuracy of model *V*_each_. However, in Treatment D, the prediction accuracy of model *V*_each_ was much higher in Week 5 than in Week 2. Therefore, MS data should be collected at different times depending on the irrigation levels of the field.

In Treatments C, W5, and W10, the prediction accuracy of model *V*_each_ was much higher than that of model NDVI_each_ using VIs other than NDVI in Week 2. In Week 5, however, these differences in the prediction accuracy were small. For yield prediction in wheat, a comparison was made between a prediction model using 62 reflectance bands in the 392–850 nm range acquired using a hyperspectral camera and one using only the single VI of NDVI (Aguate et al., 2017). Aguate et al. (2017) reported that the difference in the prediction accuracy between the two models decreased over time. Therefore, the significance of collecting MS data from multiple bands during the early stages of growth (before the flowering stage) was confirmed. In contrast, compared with the other treatments, in Treatment D, the prediction accuracy of model *V*_each_ was higher than that of model NDVI_each_ at Week 5. In Treatment D, PC1 accounted for less of the total variance in Week 5 than in the other treatments, suggesting that VIs other than NDVI were also important for prediction. Therefore, it is suggested that MS data of multiple bands should be obtained under drought conditions, even after flowering.

We expected that the growth environment would differ depending on the field heterogeneity in each condition; thus, a model with kernels **G** and **F** (*G* + Field model) was also employed. However, the prediction accuracy was almost the same as that of model *G* (Figure 6). Therefore, field heterogeneity based on simple distance did not exist in this field, and it is possible that VIs captured AGB variations that were not based on simple distances between microplots.

### 4.5 Prediction over all treatment levels

On average, the prediction accuracy of model *G* + *E* + *V*_all_ was 10% higher than that of model *G* + *E* and *G* + *E* + *GEI*. Model *G* + *E* + *V*_all_ has also been reported to have a higher prediction accuracy than model *G* + *E* + GEI for wheat yield prediction (Krause et al., 2019). In the current study, model *G* + *V*_all_ and *V*_all_ outperformed model *G* + *E* + GEI in terms of prediction accuracy after Week 4. This indicates that MS imaging can capture not only microenvironmental variability but also macroenvironmental variability, that is, the differences in irrigation levels.

Model NDVI_all_ showed almost the same prediction accuracy as model *V*_all_ in Weeks 5 and 6. In soybean, NDVI has been reported to have a high correlation with the wilting score, which is an important symptom of drought stress (Zhou et al., 2020), and AGB (Daughtry et al., 1992). Therefore, in Weeks 5 and 6 (almost after flowering), model NDVI_all_ showed high prediction accuracy, and there was no need to collect other VIs. However, from Week 1 through Week 4 (from the early vegetative to flowering stages), the prediction accuracy of model *V*_all_ was higher than that of NDVI_all_, indicating the usefulness of using multiple VIs.

### 4.6 Applicability of MS data

In this study, MS data increased the prediction accuracy of the genotypic and phenotypic values of AGB. In this experimental field, the distance between plants was >20 cm, and it was assumed that the competition between individuals was small compared with that under normal growing conditions. It has been noted that genotypes selected in such fields with small interindividual competition do not always show high performance in the actual field (Fischer & Rebetzke, 2018). In addition, in the present study, there was no replication within each treatment; thus, the observed AGB value was probably affected by nongenetic variations. In a field with replications, the prediction accuracy of the *G* model may increase, whereas the importance of MS data may decrease. Therefore, additional experiments in the field with interindividual competition and replications are necessary to clarify the absolute potential of MS data in breeding and cultivation management. However, because the significant relationship between VIs and AGB was confirmed in the present study, VIs measured before the flowering stage (and after the flowering stage) were expected to be useful for breeding and cultivation management.

## 5 CONCLUSIONS

Multispectral image data were collected from the early vegetative to the early reproductive stages of soybean, and the genetic and phenotypic correlations between VIs and the yield‐related trait AGB were determined. The results of the present study suggest that the relationship between VIs and AGB differed between treatments that were irrigated to some extent (Treatments C, W5, and W10) and those that were not irrigated (Treatment D). The results of this study will help to determine when MS data should be collected for different irrigation conditions and when only NDVI or other VIs should be collected to predict the genotypic and phenotypic values of AGB. Accumulation of this knowledge is important for breeding and cultivation management.

## AUTHOR CONTRIBUTIONS

Kengo Sakurai: Data curation; Formal analysis; Investigation; Methodology; Software; Visualization; Writing – original draft; Writing – review & editing. Yusuke Toda: Conceptualization; Data curation; Funding acquisition; Investigation. Hiromi Kajiya‐Kanegae: Resources. Yoshihiro Ohmori: Funding acquisition; Investigation. Yuji Yamasaki: Funding acquisition; Investigation. Hirokazu Takahashi: Funding acquisition; Investigation. Hideki Takanashi: Funding acquisition; Investigation. Mai Tsuda: Funding acquisition; Investigation. Hisashi Tsujimoto: Funding acquisition; Investigation. Akito Kaga: Funding acquisition; Investigation; Resources. Mikio Nakazono: Funding acquisition; Investigation. Toru Fujiwara: Funding acquisition; Investigation. Hiroyoshi Iwata: Conceptualization; Funding acquisition; Investigation; Project administration; Supervision; Writing – review & editing.

## CONFLICT OF INTEREST

The authors declare no conflict of interest.

## Supporting information


**Supplemental Figure S1**. Histogram of flowering days in each treatment.

**Supplemental Figure S2**. The flow of plant area segmentation from each microplot image.

**Supplemental Figure S3**. Aboveground biomass (AGB) prediction accuracies using additive genomic relationship kernel. G only model: without the effect for flowering, G + Flower model: with the effect for flowering in each treatment.

**Supplemental Figure S4**. Phenotypic correlations between aboveground biomass (AGB) and the vegetation indices (VIs) over all treatments.

**Supplemental Figure S5**. Heat maps for each microplot based on normalized difference vegetation index (NDVI) values calculated from multispectral (MS) data collected on Aug 31st (Week 5).

**Supplemental Table S1**. The description of accessions in each treatment.

**Supplemental Table S2**. The equations of five vegetation indices (VIs) used in this study.

**Supplemental Table S3**. Results of the analysis of variance (ANOVA) for aboveground biomass (AGB).

**Supplemental Table S4**. Results of the analysis of variance (ANOVA) for the mean of aboveground biomass (AGB) in each microplot in each treatment.

**Supplemental Table S5**. The cumulative proportion of principal component analysis (PCA) for five vegetation indices (VIs) across all irrigation levels for the different growth stages.

**Supplemental File S1**. The details of single/multiple kernel models that were used in the analysis.

## Data Availability

The datasets generated and analyzed in the present study are available from the ‘Sakuraikengo/TSMS_supple’ repository in the GitHub, https://github.com/Sakuraikengo/TSMS_supple.
