# Supplementary material for: Time‐series multispectral imaging in soybean for improving biomass and genomic prediction accuracy
Source: Plant Genome. 2022 Aug 22;15(4):e20244. doi: 10.1002/tpg2.20244 (PMC12807072; doi:10.1002/tpg2.20244)
Supplement: Supplementary file 1 [file TPG2-15-e20244-s001.docx]

Figure S4.	Phenotypic correlations between above-ground biomass (AGB) and the vegetation indices (VIs) over all treatments.
