# Supplementary material for: Time‐series multispectral imaging in soybean for improving biomass and genomic prediction accuracy
Source: Plant Genome. 2022 Aug 22;15(4):e20244. doi: 10.1002/tpg2.20244 (PMC12807072; doi:10.1002/tpg2.20244)
Supplement: Supplementary file 2 [file TPG2-15-e20244-s002.docx]

Figure S1.	Histogram of flowering days in each treatment; daily watering (control, C), no watering (drought, D), watering for 5 days followed by no watering for 5 days (W5), and watering for 10 days followed by no watering for 10 days (W10). Dashed lines represent the date of UAV measurements.
