# Supplementary material for: Time‐series multispectral imaging in soybean for improving biomass and genomic prediction accuracy
Source: Plant Genome. 2022 Aug 22;15(4):e20244. doi: 10.1002/tpg2.20244 (PMC12807072; doi:10.1002/tpg2.20244)
Supplement: Supplementary file 3 [file TPG2-15-e20244-s003.docx]

**Appendix S1	The details of single/multiple kernel models that were used in the analysis.**

**Single/Multiple Kernel Models within Each Treatment**

**The Additive genomic relationship kernel model (*G* model)**

This model predicts the phenotypic values of AGB using only genome-wide marker data. As a basis for comparison to assess the advantage of VIs, the following single-kernel regression model was built using the additive genomic relationship kernel ($\mathbf{G}$):

$$\begin{aligned} y_{il}=b_{l}+u_{i}+\varepsilon_{i}\#\left( S1 \right) \end{aligned}$$

where $y_{il}$ is the phenotypic value of AGB for genotype $i$ $\left( i=1,...,n \right)$. $b_{l}$ the fixed effect of flowering $\left( l=1,2 \right)$, $u_{i}$ the genetic random effect, and $\varepsilon_{i}$ the residual random effect. The vector $\mathbf{u}=\left( u_{1},...,u_{n} \right)^{T}$ follows the multi-variate normal distribution $MVN\left( \mathbf{0},\mathbf{G}\sigma_{g}^{2} \right)$, where $\mathbf{G}$ is the additive numerator relationship kernel and $\sigma_{g}^{2}$ is the additive genetic variance. The vector $\boldsymbol{\varepsilon}=\left( \varepsilon_{1},...,\varepsilon_{n} \right)^{T}$ follows the multi-variate normal distribution $MVN\left( \mathbf{0},\mathbf{I}\sigma_{e}^{2} \right)$, where $\mathbf{I}$ is the identical matrix and $\sigma_{e}^{2}$ is the residual variance.

**The VI relationship kernel model (**$\boldsymbol{V}_{\boldsymbol{each}}$ **model)**

To evaluate the usefulness of VIs in predicting the phenotypic values of AGB at each irrigation level, the following single-kernel model was built based on $\mathbf{V}_{each}$:

$$\begin{aligned} y_{il}=b_{l}+v_{i}+\varepsilon_{i}\#\left( S2 \right) \end{aligned}$$

where $y_{i}$, $b_{l}$, and $\varepsilon_{i}$ are defined as above in the model of Equation S1, and $v_{i}$ is the random effect of the VIs. The vector $\mathbf{v}=\left( v_{1},...,v_{n} \right)^{T}$ follows the multi-variate normal distribution $MVN\left( \mathbf{0},\mathbf{V}_{each}\sigma_{v}^{2} \right)$, where $\mathbf{V}_{each}$ is the VIs relationship kernel for each treatment and $\sigma_{v}^{2}$ is the VIs variance.

**Model with** $\mathbf{G}$ **and** $\mathbf{V}_{\boldsymbol{each}}$ **kernels (**$\boldsymbol{G}\mathbf{+}\boldsymbol{V}_{\boldsymbol{each}}$ **model)**

By using two types of kernels, the additive genomic relationship kernel ($\mathbf{G}$) and VIs relationship kernel ($\mathbf{V}_{each}$), we can make predictions using both genome and VIs data. This multi-kernel model was built using ($\mathbf{G}$) and ($\mathbf{V}_{each}$):

$$\begin{aligned} y_{il}=b_{l}+u_{i}+v_{i}+\varepsilon_{i}\#\left( S3 \right) \end{aligned}$$

Here, $y_{il}$, $b_{l}$, $u_{i}$, and $\varepsilon_{i}$ are defined as in the model of Equation S1, while $v_{i}$ is defined as in the model of Equation S2.

**Model with** $\mathbf{G}$ **kernel and field heterogeneity relationship kernel (**$\boldsymbol{G}\mathbf{+}\boldsymbol{Field}$ **model)**

To confirm that there was a field heterogeneity resulted in the distances among plots or not in the experimental field. This multi-kernel model was built using additive genomic relationship kernel ($\mathbf{G}$) and the field heterogeneity relationship kernel ($\mathbf{FH}$):

$$\begin{aligned} y_{il}=b_{l}+u_{i}+f_{i}+\varepsilon_{i}\#\left( S4 \right) \end{aligned}$$

Here, $y_{il}$, $b_{l}$, $u_{i}$, and $\varepsilon_{i}$ are defined as in the model of Equation S1, and $f_{i}$ is the random effect reflecting the among-plot distances. The vector $\mathbf{f}=\left( f_{1},...,f_{n} \right)^{T}$ follows the multi-variate normal distribution $MVN\left( \mathbf{0},\mathbf{FH}\sigma_{f}^{2} \right)$, where $\mathbf{FH}$ is the field heterogeneity relationship matrix and $\sigma_{f}^{2}$ is the variance of field heterogeneity based on the among-plot distances.

**Single/Multiple Kernel Models over All Treatments**

**Model with** $\mathbf{G}$ **kernel and treatment (**$\boldsymbol{G}\mathbf{+}\boldsymbol{E}$ **model)**

This model assumes that genome-wide marker data and treatment information are available:

$$\begin{aligned} y_{ijl}=E_{j}+b_{l}+u_{i}+\varepsilon_{ij}\#\left( S5 \right) \end{aligned}$$

Here, $u_{i}$ $\left( i=1,...,n \right)$is defined as above in the model of Equation S1, $y_{ij}$ is the phenotypic trait value of AGB for genotype $i$ in treatment $j$, $E_{j}$ is the fixed effect for treatment $j$ $\left( j=1,...,m \right)$, $b_{l}$ is the fixed effect of flowering or not $\left( l=1,2 \right)$, and $\varepsilon_{ij}$ is the residual random effect. $\boldsymbol{\varepsilon}=\left( \varepsilon_{11},...,\varepsilon_{nm} \right)^{T}$ follows the multi-normal distribution $MVN\left( \mathbf{0},\mathbf{I}\sigma_{e}^{2} \right)$, where $\mathbf{I}$ is the identical matrix and, $\sigma_{e}^{2}$ is the residual variance.

**Model with** $\mathbf{G}$ **kernel and treatment and genotype-by-treatment interaction effects (**$\boldsymbol{G}\mathbf{+}\boldsymbol{E}\mathbf{+}\boldsymbol{G}\boldsymbol{\times}\boldsymbol{E}$ **model)**

The effects of treatment and genotype-by-environment interaction were added to the $G+E$ model:

$$\begin{aligned} y_{ijl}=E_{j}+b_{l}+u_{i}+ge_{ij}+\varepsilon_{ij}\#\left( S6 \right) \end{aligned}$$

Here, $y_{ijl}$, $E_{j}$, $b_{l}$, $u_{i}$, and $\varepsilon_{ij}$ are defined as above in model Equation S5. $ge_{ij}$ is the genotype-by-environment interaction random effect. $\mathbf{ge}=\left( ge_{11},...,ge_{nm} \right)^{T}$ follows multi-normal distribution $MVN\left( \mathbf{0},\left( {\mathbf{Z}_{\mathbf{g}}\mathbf{G}\mathbf{Z}_{\mathbf{g}}}^{T} \right)\circ\left( {\mathbf{Z}_{\mathbf{E}}\mathbf{Z}_{\mathbf{E}}}^{T} \right)\sigma_{ge}^{2} \right)$, where $\mathbf{Z}_{\mathbf{g}}$ and $\mathbf{Z}_{\mathbf{E}}$ are incidence matrices for genotypes and treatments, and $\sigma_{ge}^{2}$ is the variance component for the effect of genotype-by-environment interaction (Jarquín et al., 2014; Pérez-Rodríguez et al., 2015; Pérez‐Rodríguez et al., 2017).

**Model with** $\mathbf{G}$ **and** $\mathbf{V}_{\boldsymbol{all}}$ **kernels and the fixed effect of treatment (**$\boldsymbol{G}\mathbf{+}\boldsymbol{E}\mathbf{+}\boldsymbol{V}_{\boldsymbol{all}}$ **model)**

To model the $G\times E$ effect using the VIs relationship kernel ($\mathbf{V}_{all}$), this multi-kernel model includes genome-wide marker data, treatment information, and phenotypic traits of VIs:

$$\begin{aligned} y_{ijl}=E_{j}+b_{l}+u_{i}+v_{ij}+\varepsilon_{ij}\#\left( S7 \right) \end{aligned}$$

Here, $y_{ijl}$, $E_{j}$, $b_{l}$, $u_{i}$, and $\varepsilon_{ij}$ are defined as above in model Equation S5. $v_{ij}$ is the random effect of the VIs. The vector $\mathbf{v}=\left( v_{11},...,v_{nm} \right)^{T}$ follows the multi-variate normal distribution $\mathbf{v}\sim MVN\left( \mathbf{0},\mathbf{V}_{all}\sigma_{v}^{2} \right)$, where $\mathbf{V}_{all}$ is the VIs relationship kernel over all treatment and $\sigma_{v}^{2}$ is the VIs variance.

**Model with** $\mathbf{G}$ **and** $\mathbf{V}_{\boldsymbol{all}}$ **kernels (**$\boldsymbol{G}\mathbf{+}\boldsymbol{V}_{\boldsymbol{all}}$ **model)**

This model evaluates whether the VIs can capture the macro-environmental variability, i.e., the differences in irrigation levels. This model predicts the phenotypic values of AGB from marker information and the phenotypic data of VIs without treatment information. This model was nearly the same for Equation S3:

$$\begin{aligned} y_{ijl}=b_{l}+u_{i}+v_{ij}+\varepsilon_{ij}\#\left( S8 \right) \end{aligned}$$

Here, $y_{ij}$, $b_{l}$, $u_{i}$, and $\varepsilon_{ij}$ are defined as above in Equation S5. $v_{ij}$ is defined as above in Equation S6.

**Model with** $\mathbf{V}_{\boldsymbol{all}}$ **kernels (**$\boldsymbol{V}_{\boldsymbol{all}}$ **model)**

This model was built using only VIs phenotypic data, considering the that neither genome-wide marker data nor treatment information were available. This model was almost the same for Equation S2:

$$\begin{aligned} y_{ijl}=b_{l}+v_{ij}+\varepsilon_{ij}\#\left( S9 \right) \end{aligned}$$

Here, $y_{ijl}$, $b_{l}$, and $\varepsilon_{ij}$ are defined as above in model Equation S5. $v_{ij}$ is defined as above in model Equation S6.

References

Chen, P. F., Nicolas, T., Wang, J. H., Philippe, V., Huang, W. J., & Li, B. G. (2010). New index for crop canopy fresh biomass estimation. Spectroscopy and Spectral Analysis, 30(2), 512-517.

Delegido, J., Verrelst, J., Meza, C. M., Rivera, J. P., Alonso, L., & Moreno, J. (2013). A red-edge spectral index for remote sensing estimation of green LAI over agroecosystems. *European Journal of Agronomy*, *46*, 42–52. https://doi.org/10.1016/j.eja.2012.12.001

Jarquín, D., Crossa, J., Lacaze, X., Du Cheyron, P., Daucourt, J., Lorgeou, J., Piraux, F., Guerreiro, L., Pérez, P., Calus, M., Burgueño, J., & de los Campos, G. (2014). A reaction norm model for genomic selection using high-dimensional genomic and environmental data. *Theoretical and Applied Genetics*, *127*(3), 595–607. https://doi.org/10.1007/s00122-013-2243-1

Jorge, J., Vallbé, M., & Soler, J. A. (2019). Detection of irrigation inhomogeneities in an olive grove using the NDRE vegetation index obtained from UAV images. *European Journal of Remote Sensing*, *52*(1), 169–177. https://doi.org/10.1080/22797254.2019.1572459

Motohka, T., Nasahara, K. N., Oguma, H., & Tsuchida, S. (2010). Applicability of Green-Red Vegetation Index for remote sensing of vegetation phenology. *Remote Sensing*, *2*(10), 2369–2387. https://doi.org/10.3390/rs2102369

Pérez-Rodríguez, P., Crossa, J., Bondalapati, K., De Meyer, G., Pita, F., & De Los Campos, G. (2015). A pedigree-based reaction norm model for prediction of cotton yield in multienvironment trials. *Crop Science*, *55*(3), 1143–1151. https://doi.org/10.2135/cropsci2014.08.0577

Pérez‐Rodríguez, P., Crossa, J., Rutkoski, J., Poland, J., Singh, R., Legarra, A., Autrique, E., Campos, G. de los, Burgueño, J., & Dreisigacker, S. (2017). Single‐Step Genomic and Pedigree Genotype × Environment Interaction Models for Predicting Wheat Lines in International Environments. *The Plant Genome*, *10*(2). https://doi.org/10.3835/plantgenome2016.09.0089

Price, J. C., & Bausch, W. C. (1995). Leaf area index estimation from visible and near-infrared reflectance data. *Remote Sensing of Environment*, *52*(1), 55–65. https://doi.org/10.1016/0034-4257(94)00111-Y

Zarate-Valdez, J. L., Metcalf, S., Stewart, W., Ustin, S. L., & Lampinen, B. (2015). Potentials and limits of vegetation indices for LAI and APAR assessment. *Precision Agriculture.*, *16*(4), 161–173. https://doi.org/10.2136/sssaj1977.03615995004100040037x
