# Supplementary material for: Time‐series multispectral imaging in soybean for improving biomass and genomic prediction accuracy
Source: Plant Genome. 2022 Aug 22;15(4):e20244. doi: 10.1002/tpg2.20244 (PMC12807072; doi:10.1002/tpg2.20244)
Supplement: Supplementary file 4 [file TPG2-15-e20244-s004.docx]

**Table S4**	**Results of the analysis of variance (ANOVA) for the mean of above-ground biomass (AGB) in each microplot in each treatment; daily watering (control, C), no watering (drought, D), watering for 5 days followed by no watering for 5 days (W5), and watering for 10 days followed by no watering for 10 days (W10).**

|  | **Treatment** | **Variable** | **Df** | **Sum Sq** | **F-value** | **P-value** |
| --- | --- | --- | --- | --- | --- | --- |
| Week2 | C | Flowering | 1 | 9066.13596 | 24.3531832 | **1.8e-06*** |
|  |  | Residuals | 188 | 69988.1221 |  |  |
|  | W5 | Flowering | 1 | 776.31982 | 3.6781728 | 0.057 |
|  |  | Residuals | 188 | 39679.5186 |  |  |
|  | W10 | Flowering | 1 | 570.151322 | 3.68524128 | 0.056 |
|  |  | Residuals | 188 | 29085.8699 |  |  |
|  | D | Flowering | 1 | 2.56410308 | 0.10481652 | 0.75 |
|  |  | Residuals | 181 | 4427.76253 |  |  |
| Week3 | C | Flowering | 1 | 5431.00032 | 13.8682815 | **0.00026*** |
|  |  | Residuals | 188 | 73623.2577 |  |  |
|  | W5 | Flowering | 1 | 1719.42703 | 8.34492071 | 0.0043 |
|  |  | Residuals | 188 | 38736.4114 |  |  |
|  | W10 | Flowering | 1 | 844.099687 | 5.50781526 | 0.020 |
|  |  | Residuals | 188 | 28811.9216 |  |  |
|  | D | Flowering | 1 | 84.3176156 | 3.51160993 | 0.063 |
|  |  | Residuals | 181 | 4346.00902 |  |  |
| Week4 | C | Flowering | 1 | 7129.62959 | 18.6357635 | **2.6e-05*** |
|  |  | Residuals | 188 | 71924.6285 |  |  |
|  | W5 | Flowering | 1 | 1330.99397 | 6.39560028 | 0.012 |
|  |  | Residuals | 188 | 39124.8445 |  |  |
|  | W10 | Flowering | 1 | 316.740672 | 2.02960826 | 0.16 |
|  |  | Residuals | 188 | 29339.2806 |  |  |
|  | D | Flowering | 1 | 139.947891 | 5.90404013 | 0.016 |
|  |  | Residuals | 181 | 4290.37874 |  |  |
| Week5 | C | Flowering | 1 | 371.841246 | 0.88845967 | 0.35 |
|  |  | Residuals | 188 | 78682.4168 |  |  |
|  | W5 | Flowering | 1 | 5.46242495 | 0.02538755 | 0.87 |
|  |  | Residuals | 188 | 40450.376 |  |  |
|  | W10 | Flowering | 1 | 5.36376066 | 0.03400893 | 0.85 |
|  |  | Residuals | 188 | 29650.6575 |  |  |
|  | D | Flowering | 1 | 86.4362881 | 3.60160292 | 0.059 |
|  |  | Residuals | 181 | 4343.89034 |  |  |
| Week6 | C | Flowering | 1 | 30.670647 | 0.07296659 | 0.79 |
|  |  | Residuals | 188 | 79023.5874 |  |  |
|  | W5 | Flowering | 1 | 96.6964086 | 0.45042892 | 0.50 |
|  |  | Residuals | 188 | 40359.142 |  |  |
|  | W10 | Flowering | 1 | 1172.3948 | 7.73813765 | 0.0060 |
|  |  | Residuals | 188 | 28483.6265 |  |  |
|  | D | Flowering | 1 | 67.5373869 | 2.80193847 | 0.096 |
|  |  | Residuals | 181 | 4362.78924 |  |  |

Degrees of freedom (Df); Sum square (Sum Sq); Mean square (Mean Sq)

* indicate that p < 0.001
