# Supplementary material for: Time‐series multispectral imaging in soybean for improving biomass and genomic prediction accuracy
Source: Plant Genome. 2022 Aug 22;15(4):e20244. doi: 10.1002/tpg2.20244 (PMC12807072; doi:10.1002/tpg2.20244)
Supplement: Supplementary file 5 [file TPG2-15-e20244-s005.docx]

**Table S2**	**The equations of five vegetation indices (VIs) which are used in this study.**

| **Index** | **Equation** | **References** |
| --- | --- | --- |
| Normalized difference vegetation index (NDVI) | $(\rho850-\rho660)/$  $(\rho850+\rho660)$ | (Price & Bausch, 1995; Zarate-Valdez et al., 2015) |
| Normalized difference red-edge index (NDRE) | $(\rho850-\rho725)/$  $(\rho850+\rho725)$ | (Jorge et al., 2019) |
| Green red vegetation index (GRVI) | $({\rho550}_{1}-\rho660)/$  $({\rho550}_{1}+\rho660)$ | (Motohka et al., 2010) |
| Normalized difference index using red-edge and red spectral reflectance (NDI_red_) | $(\rho725-\rho660)/$  $(\rho725+\rho660)$ | (Delegido et al., 2013) |
| Red-edge triangular vegetation index (RTVI) | $(\rho850-\rho725)-$  $10(\rho850-\rho550)$ | (Chen et al., 2010) |

Note: $\rho660, \rho725,$ etc. represent the spectral reflectance of 660 nm and 725 nm. ${\rho550}_{1}$ represents the spectral reflectance of 550 nm collected from TB550/660/850. $\rho550$ represent the average of spectral reflectance of 550 nm collected from TB475/550/850 and TB550/660/850. $\rho850$ represent the average of spectral reflectance of 850 nm collected from TB475/550/850 and TB550/660/850.
