# Supplementary material for: Time‐series multispectral imaging in soybean for improving biomass and genomic prediction accuracy
Source: Plant Genome. 2022 Aug 22;15(4):e20244. doi: 10.1002/tpg2.20244 (PMC12807072; doi:10.1002/tpg2.20244)
Supplement: Supplementary file 7 [file TPG2-15-e20244-s007.docx]

**Table S5**	**The cumulative proportion of principal component analysis (PCA) for five vegetation indices (VIs) across all drought treatments for the different growth stages.**

|  | PC1 | PC2 | PC3 | PC4 | PC5 |
| --- | --- | --- | --- | --- | --- |
| Week1 | 0.5 | 0.85 | 0.98 | 0.99 | 1 |
| Week2 | 0.56 | 0.94 | 0.99 | 1 | 1 |
| Week3 | 0.77 | 0.97 | 0.99 | 1 | 1 |
| Week4 | 0.81 | 0.98 | 1 | 1 | 1 |
| Week5 | 0.88 | 0.98 | 1 | 1 | 1 |
| Week6 | 0.9 | 0.98 | 1 | 1 | 1 |
