# Supplementary material for: Time‐series multispectral imaging in soybean for improving biomass and genomic prediction accuracy
Source: Plant Genome. 2022 Aug 22;15(4):e20244. doi: 10.1002/tpg2.20244 (PMC12807072; doi:10.1002/tpg2.20244)
Supplement: Supplementary file 8 [file TPG2-15-e20244-s008.docx]

Figure S2.	The flow of plant area segmentation from each plot image. (a) Image of each plot, colored with a gradient according to the normalized difference vegetation index (NDVI) value; the plant area is within the green frame, the white mulching sheet area is within the blue frame, and the soil area is within the red frame. (b) Histogram of NDVI value within each color frame in (a); each color corresponds to the respective color of the frame. (c) Image masked with NDVI threshold of 0.15.
