# Supplementary material for: Time‐series multispectral imaging in soybean for improving biomass and genomic prediction accuracy
Source: Plant Genome. 2022 Aug 22;15(4):e20244. doi: 10.1002/tpg2.20244 (PMC12807072; doi:10.1002/tpg2.20244)
Supplement: Supplementary file 9 [file TPG2-15-e20244-s009.docx]

**Table S3**	**Results of the analysis of variance (ANOVA) for above-ground biomass (AGB).**

|  | **Df** | **Sum Sq** | **Mean Sq** | **F-value** | **P-value** |
| --- | --- | --- | --- | --- | --- |
| Treatment | 3 | 293053 | 97684 | 502.8067 | < **2.2e-16*** |
| Accession | 193 | 137893 | 714 | 3.6776 | < **2.2e-16*** |
| Residuals | 1296 | 251784 | 194 |  |  |

Degrees of freedom (Df); Sum square (Sum Sq); Mean square (Mean Sq)

* indicate that p < 0.001
