# Supplementary material for: Time‐series multispectral imaging in soybean for improving biomass and genomic prediction accuracy
Source: Plant Genome. 2022 Aug 22;15(4):e20244. doi: 10.1002/tpg2.20244 (PMC12807072; doi:10.1002/tpg2.20244)
Supplement: Supplementary file 10 [file TPG2-15-e20244-s010.docx]

Figure S5.	Heat maps for each micro plot based on NDVI values calculated from MS data collected on Aug 31st (Week 5). (a) Heat map of NDVI values for a particular micro plot in treatment C. (b) Heatmap of NDVI values for a particular micro plot in treatment D. (c) The figure of plant body segmentation with NDVI threshold of 0.15 based on the image in (a). (d) The figure of plant body segmentation with NDVI threshold of 0.15 based on the image in (b). These values that are above of (c) and (d) are the number of pixels that are considering belonging to plants.
