# Supplementary material for: Time‐series multispectral imaging in soybean for improving biomass and genomic prediction accuracy
Source: Plant Genome. 2022 Aug 22;15(4):e20244. doi: 10.1002/tpg2.20244 (PMC12807072; doi:10.1002/tpg2.20244)
Supplement: Supplementary file 11 [file TPG2-15-e20244-s011.docx]

**Table S1	The description of accessions in each treatment; daily watering (control, C), no watering (drought, D), watering for 5 days followed by no watering for 5 days (W5), and watering for 10 days followed by no watering for 10 days (W10). We used 198 accessions of soybean genetic resources, registered as the mini core collections in the National Institute of Agrobiological Sciences (NIAS) gene bank. Some accessions were removed due to the unavailability of phenotypic traits. NAs represent unavailable phenotypic data, while blanks represent data used in this study.**

| **Accession** | **Treatment C** | **Treatment W5** | **Treatment W10** | **Treatment D** |
| --- | --- | --- | --- | --- |
| GmJMC002 |  |  |  | NA |
| GmJMC003 |  |  |  |  |
| GmJMC004 |  |  |  |  |
| GmJMC005 |  |  |  |  |
| GmJMC007 |  |  |  |  |
| GmJMC008 |  |  |  |  |
| GmJMC009 |  |  |  |  |
| GmJMC013 |  |  |  |  |
| GmJMC016 |  |  |  |  |
| GmJMC017 |  |  |  |  |
| GmJMC021 |  |  |  |  |
| GmJMC023 |  |  |  |  |
| GmJMC025 |  |  |  |  |
| GmJMC026 |  |  |  |  |
| GmJMC028 |  |  |  |  |
| GmJMC030 |  |  |  |  |
| GmJMC031 |  |  |  |  |
| GmJMC032 |  |  |  |  |
| GmJMC033 |  |  |  |  |
| GmJMC034 |  |  |  |  |
| GmJMC037 |  |  |  |  |
| GmJMC039 |  |  |  |  |
| GmJMC040 |  |  |  |  |
| GmJMC041 |  |  |  |  |
| GmJMC043 |  |  |  |  |
| GmJMC044 |  |  |  |  |
| GmJMC047 |  |  |  |  |
| GmJMC049 |  |  |  |  |
| GmJMC050 |  |  |  |  |
| GmJMC051 |  |  |  |  |
| GmJMC052 |  |  |  |  |
| GmJMC053 |  |  |  |  |
| GmJMC054 |  |  |  | NA |
| GmJMC055 |  |  |  |  |
| GmJMC056 |  |  | NA | NA |
| GmJMC057 |  |  |  |  |
| GmJMC058 |  |  |  |  |
| GmJMC059 |  |  |  |  |
| GmJMC060 |  |  |  |  |
| GmJMC061 |  |  |  |  |
| GmJMC062 |  |  |  |  |
| GmJMC063 |  |  |  |  |
| GmJMC064 |  |  |  |  |
| GmJMC065 |  |  |  |  |
| GmJMC067 |  |  |  |  |
| GmJMC068 |  |  |  |  |
| GmJMC069 |  |  |  |  |
| GmJMC076 |  |  |  |  |
| GmJMC077 |  |  |  |  |
| GmJMC078 |  |  |  |  |
| GmJMC079 |  |  |  |  |
| GmJMC080 |  |  |  |  |
| GmJMC081 |  |  | NA | NA |
| GmJMC082 |  |  |  |  |
| GmJMC085 |  |  |  |  |
| GmJMC088 |  | NA |  |  |
| GmJMC090 | NA | NA |  |  |
| GmJMC091 |  |  |  |  |
| GmJMC092 |  |  |  |  |
| GmJMC093 |  |  |  |  |
| GmJMC095 |  |  |  | NA |
| GmJMC096 |  |  |  |  |
| GmJMC097 |  |  |  |  |
| GmJMC098 |  |  |  | NA |
| GmJMC099 |  |  |  |  |
| GmJMC100 |  |  |  |  |
| GmJMC101 |  |  |  |  |
| GmJMC102 |  |  |  |  |
| GmJMC104 |  |  |  |  |
| GmJMC105 |  |  | NA |  |
| GmJMC106 |  |  |  | NA |
| GmJMC110 |  |  |  |  |
| GmJMC111 |  |  |  | NA |
| GmJMC112 |  |  |  |  |
| GmJMC114 |  |  | NA |  |
| GmJMC116 |  |  |  |  |
| GmJMC117 |  |  |  |  |
| GmJMC121 |  |  |  |  |
| GmJMC126 |  |  |  |  |
| GmJMC128 |  |  |  |  |
| GmJMC130 |  |  |  |  |
| GmJMC131 |  |  |  |  |
| GmJMC133 |  |  |  |  |
| GmJMC137 |  |  |  |  |
| GmJMC139 |  |  |  |  |
| GmJMC145 |  |  |  |  |
| GmJMC149 |  |  |  |  |
| GmJMC158 |  |  |  | NA |
| GmJMC161 |  |  |  |  |
| GmJMC167 |  |  |  |  |
| GmJMC172 |  |  |  |  |
| GmJMC177 |  |  |  |  |
| GmJMC179 |  |  |  |  |
| GmJMC180 |  |  |  |  |
| GmJMC184 |  |  |  |  |
| GmWMC001 |  |  |  |  |
| GmWMC006 |  |  |  |  |
| GmWMC010 |  |  |  |  |
| GmWMC011 |  |  |  |  |
| GmWMC012 |  |  |  |  |
| GmWMC014 |  |  |  |  |
| GmWMC015 |  |  |  |  |
| GmWMC018 | NA |  |  |  |
| GmWMC019 |  |  |  |  |
| GmWMC020 | NA | NA | NA | NA |
| GmWMC022 |  |  |  |  |
| GmWMC024 |  |  |  |  |
| GmWMC027 |  |  |  |  |
| GmWMC029 |  |  |  |  |
| GmWMC035 |  |  |  |  |
| GmWMC036 |  |  |  |  |
| GmWMC038 | NA |  |  |  |
| GmWMC042 |  |  |  |  |
| GmWMC045 |  |  |  |  |
| GmWMC046 |  |  |  |  |
| GmWMC048 |  |  |  |  |
| GmWMC066 |  |  |  |  |
| GmWMC070 |  |  |  |  |
| GmWMC071 |  |  |  |  |
| GmWMC072 |  |  |  |  |
| GmWMC073 |  |  |  |  |
| GmWMC074 |  |  |  |  |
| GmWMC075 |  |  |  |  |
| GmWMC083 |  |  |  |  |
| GmWMC084 |  |  |  |  |
| GmWMC086 |  |  |  |  |
| GmWMC087 |  | NA |  |  |
| GmWMC089 |  |  |  |  |
| GmWMC094 |  |  |  |  |
| GmWMC103 |  |  |  |  |
| GmWMC107 |  |  |  |  |
| GmWMC108 |  |  |  |  |
| GmWMC109 |  |  |  |  |
| GmWMC113 | NA | NA |  |  |
| GmWMC115 |  |  |  |  |
| GmWMC118 |  |  |  |  |
| GmWMC119 |  |  |  |  |
| GmWMC120 |  |  |  |  |
| GmWMC122 |  |  |  |  |
| GmWMC123 |  |  |  |  |
| GmWMC124 |  |  |  |  |
| GmWMC125 |  |  |  |  |
| GmWMC127 |  |  |  |  |
| GmWMC129 |  |  |  |  |
| GmWMC132 |  |  |  |  |
| GmWMC134 |  |  |  |  |
| GmWMC135 |  |  |  |  |
| GmWMC136 |  |  |  |  |
| GmWMC138 |  |  |  | NA |
| GmWMC140 |  |  |  |  |
| GmWMC141 |  |  |  |  |
| GmWMC142 |  |  |  |  |
| GmWMC143 |  |  |  |  |
| GmWMC144 |  |  |  |  |
| GmWMC146 |  |  |  |  |
| GmWMC147 |  |  |  |  |
| GmWMC148 |  |  |  |  |
| GmWMC150 |  |  |  |  |
| GmWMC151 |  |  |  |  |
| GmWMC152 |  |  |  |  |
| GmWMC153 |  |  |  |  |
| GmWMC154 |  |  |  |  |
| GmWMC155 |  |  |  |  |
| GmWMC156 |  |  |  | NA |
| GmWMC157 |  |  |  |  |
| GmWMC159 |  |  |  |  |
| GmWMC160 |  |  |  |  |
| GmWMC162 |  |  |  |  |
| GmWMC163 |  |  |  |  |
| GmWMC164 |  |  |  |  |
| GmWMC165 |  |  |  |  |
| GmWMC166 |  |  |  |  |
| GmWMC168 |  |  |  |  |
| GmWMC169 |  |  |  |  |
| GmWMC170 |  |  |  |  |
| GmWMC171 |  |  |  |  |
| GmWMC173 |  |  |  |  |
| GmWMC174 |  |  |  |  |
| GmWMC175 |  |  |  |  |
| GmWMC176 |  |  |  |  |
| GmWMC178 |  |  |  |  |
| GmWMC181 |  |  |  |  |
| GmWMC182 |  |  |  |  |
| GmWMC183 |  |  |  |  |
| GmWMC185 |  |  |  |  |
| GmWMC186 |  |  |  |  |
| GmWMC187 |  |  |  |  |
| GmWMC188 |  |  |  |  |
| GmWMC189 |  |  |  |  |
| GmWMC190 |  |  |  |  |
| GmWMC191 |  |  |  |  |
| GmWMC192 |  |  |  |  |
| Houjaku Kuwazu |  |  |  |  |
| Misuzudaizu | NA | NA | NA | NA |
| Norin2 | NA | NA | NA | NA |
| 5002T |  |  |  |  |
| C1329 |  |  |  |  |
| B01167 | NA | NA | NA | NA |
